# Prion replication in organotypic brain slice cultures is distinct from in vivo inoculation and is species dependent

**DOI:** 10.1186/s40478-025-01999-w

**Published:** 2025-04-30

**Authors:** Jessy A. Slota, Lise Lamoureux, Jennifer Myskiw, Kathy L. Frost, Sarah J. Medina, Dominic M. S. Kielich, Melanie Leonhardt, Gunjan Thapar, Ben A. Bailey-Elkin, Stephanie A. Booth

**Affiliations:** 1 Mycobacteriology, Vector-Borne and Prion Diseases Division, National Microbiology Laboratory, Public Health Agency of Canada, Winnipeg, MB Canada; 2 Department of Medical Microbiology and Infectious Diseases, Faculty of Health Sciences, University of Manitoba, Winnipeg, MB Canada

## Abstract

Cultured brain slices rapidly replicate murine prions, exhibit prion pathology, and are amenable towards drug discovery, but have not been infected with human prions. As deer mice (*Peromyscus maniculatus*) are susceptible to human prions in vivo, here we investigated deer mouse organotypic brain slice cultures as a potential model of human prion disease. Deer mouse brain slices supported replication of rodent-adapted strains of scrapie and Creutzfeldt-Jakob disease (CJD), but they resisted infection with primary human prion inoculum. To better understand this discrepancy, we quantified prion replication rates, characterized cellular and molecular changes, and estimated inoculum clearance within wildtype CD1 and deer mouse brain slice cultures. Prion replication rates varied by species, strain, and brain region, independently of PrP sequence homology. Scrapie-infected CD1 cerebellar slice cultures exhibited the fastest prion replication rate, closely matching in vivo bioassay kinetics and showing neuronal and synaptic degeneration at similar timepoints. However, deer mouse slice cultures replicated deer mouse-adapted sCJD MM1 prions less efficiently than in vivo inoculation. These findings clarify both the utility and constraints of brain slice cultures in modeling prion disease and imply that the slice culture molecular environment may be suboptimal for human prion replication.

**Supplementary Information:**

The online version contains supplementary material available at 10.1186/s40478-025-01999-w.

## Introduction

Prion diseases are rare, fatal, infectious neurodegenerative disorders characterized by rapid progression [1]. Sporadic Creutzfeldt-Jakob disease (sCJD) is the most common prion disease afflicting humans (85% of cases), followed by familial (e.g. fCJD) and transmitted (e.g. iCJD) forms [2, 3]. Variably protease-sensitive prionopathy (VPSPr), a rare sporadic prion disease (2% of cases), is distinguished by unusual proteinase-K sensitive PrP^Sc^ [4]. Animal prion diseases include scrapie in sheep, bovine spongiform encephalopathy (BSE) in cattle, and chronic wasting disease (CWD) in cervids. The infectious agent, or’prion’, consists of misfolded prion proteins (PrP^Sc^), which convert normal cellular prion proteins (PrP^C^) into the disease-associated structure, thereby’seeding’ prion self-assembly into amyloid fibrils [5]. Prion deposition in the brain leads to spongiosis, reactive gliosis, neuronal demise, cognitive decline and eventual death [6].

Prion disease research relies on diverse experimental models, from cell-free conversion assays [7, 8] to cell culture models and full scale animal studies, with rodent bioassays considered the ‘gold standard’ prion model for mimicking natural prion diseases [9]. Some rodent models are susceptible human prions, like transgenic human-PrP^C^-expressing mice [10] and bank voles [11]. Similarly, we recently identified the deer mouse (*Peromyscus maniculatus*) as highly susceptible to sCJD infection in vivo (J. Myskiw et al., manuscript in preparation). Deer mice are also susceptible to CWD prions [12] and share a PrP^C^ amino-acid sequence highly similar to bank vole, deviating by only two C-terminal residues (Supplementary Fig. 1) [13–15]. This similarity suggests that deer mouse PrP, like bank vole PrP [11], may also misfold when seeded by prions from multiple species, including humans.

While animal prion bioassays are valuable, they are lengthy, laborious, and costly. Cell-based prion infection models offer fast, accessible, scalable, and high-throughput alternatives while reducing animal usage [16–23]. Some cellular models, like human cerebral organoids derived from induced pluripotent stem cells [24–26], have been reported to support CJD infection [27–30]. However, choosing an appropriate cell model remains challenging since prion susceptibility must be determined empirically. Additionally, most studies report detection of prions without in-depth quantifications of prion replication, limiting direct comparisons of prion susceptibilities across paradigms.

Organotypic brain slice cultures serve as an intermediate prion model between cell cultures and animal bioassays and are thought to preserve brain morphology, cellular connectivity, and microenvironment for weeks to months in vitro [31]. This prion organotypic slice culture assay (POSCA) reproduces prion neuropathology following exponential prion replication [32–34] and has been used to study rodent-adapted scrapie strains (RML, ME7, 22L, and 139A), the 301C BSE strain, and CWD prions [32–36]. POSCA’s apparent physiological faithfulness is considered promising for therapeutic screening, but key limitations remain. Brain slice cultures have yet to be infected with human prions, constraining POSCA’s clinical relevance. Additionally, POSCA studies often use transgenic mice overexpressing PrP^C^ (e.g. Tga20) [32, 34, 36, 37] rather than wildtype mice with physiological expression levels. While wildtype cerebellar brain slices support prion replication [34, 35, 37], neuronal toxicity remains unconfirmed. Lastly, the relative susceptibility of cultured brain slices compared to animal models is unclear, necessitating direct comparisons to assess how well POSCA recapitulates prion disease.

Here, we evaluated how well POSCA reproduces prion disease in wildtype CD1 and deer mouse brain slice cultures. We initially set out to explore whether deer mouse brain slices were susceptible to both mouse and human prion inoculum. First, we compared RML scrapie infection in CD1 (an established model) and deer mouse cerebellar and whole brain slice cultures, confirming prion replication in deer mouse slices. We then tested human and rodent-adapted sCJD inocula in deer mouse slices, finding that while deer mouse-adapted sCJD MM1 prions replicated, all human prions tested (sCJD MM1, sCJD VV2, VPSPr) failed to establish infection, contrasting with in vivo results. Therefore, to investigate this discrepancy, we quantified prion replication rates, characterized cellular and molecular changes, and estimated inoculum clearance within wildtype brain slice cultures. Kinetic analysis revealed that prion replication rates in POSCA vary by prion strain, species, and brain region. RML-seeded CD1 cerebellar slices closely matched in vivo bioassays, evidenced by prion replication kinetics and onset of neuronal degeneration. However, prion replication of deer mouse-adapted sCJD MM1 prions was slower in deer mouse slices compared to in vivo inoculation, suggesting differences in molecular environment rather than PrP sequence homology. These findings highlight both the utility and limitations of POSCA for studying prion disease. While deer mouse slice cultures successfully replicated rodent-adapted prions, their resistance to human prions despite in vivo susceptibility underscores challenges in modeling human prion disease in vitro.

## Results

### CD1 cerebellar and whole brain slice cultures support prion infection

Implementing POSCA is challenging because slice cultures of suboptimal quality may not remain viable for the extended culture period needed to accumulate prion infectivity (i.e. > 42 days). This raises the possibility that different research groups may produce slice cultures of differing quality, which could influence the results reported from POSCA experiments. To demonstrate the quality of the CD1 and deer mouse cerebellar and whole brain slice cultures used in this study, we present some initial pilot experiments used to optimize POSCA in the supplementary information. We examined parameters including slice thickness (Supplementary Fig. 2) and inoculation method (Supplementary Fig. 3). Unless indicated otherwise, here we used 300 μm cerebellar sections and 200 μm whole brain sections that were inoculated via direct application of diluted brain homogenates to slices one day after culturing.

**Fig. 1:**
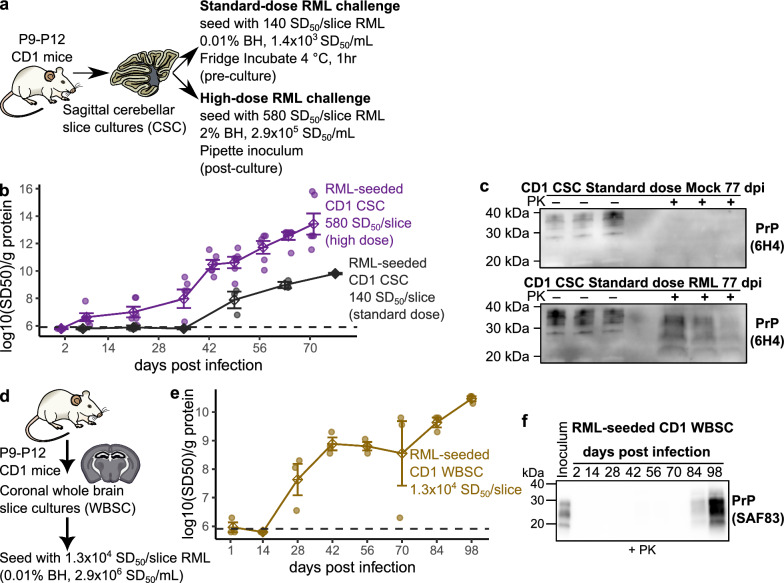
CD1 cerebellar and whole brain slice cultures support prion infection. **a** CD1 cerebellar slice cultures were seeded with either a standard dose (0.01% brain homogenate [BH], 1.4 × 10^3^ SD_50_/mL, pre-culture) or a high dose (2% BH, 2.9 × 10^5^ SD_50_/mL, post-culture) of RML scrapie. **b** PrP^Sc^ amyloid seeding activity accumulated over multiple timepoints post-challenge for each dose. **c** Proteinase K-resistant PrP^Sc^ was detected in prion-infected cerebellar slice cultures at 11 weeks post-infection (wpi) in cultures exposed to the standard-dose challenge. **d** Coronal whole brain slice cultures from CD1 mice were seeded with 1.3 × 10^4^ SD_50_ units of RML inoculum (post-culture) and productively accumulated both **(e)** PrP^Sc^ seeding activity **(f)** and proteinase K-resistant PrP^Sc^ over 98 dpi

To verify that our slice cultures were of sufficient quality to support prion infection, we next quantified prion replication in relation to inoculum dosage. We compared CD1 cerebellar slice cultures challenged with a standard-dose (0.01% brain homogenate) and high-dose (2% brain homogenate) of RML scrapie inoculum (Fig. 1). Prion replication curves were generated by measuring prion seeding activity every 14 days for up to 70 days post infection (Fig. 1b and Supplementary Fig. 4). As expected, the high-dose challenge resulted in faster prion accumulation, with seeding activity detected as early as 21 dpi compared to first detection at 49 dpi for the standard dose challenge. To confirm prion replication, we characterized proteinase K resistant PrP^Sc^ at 77 dpi following the standard-dose inoculation (Fig. 1c and Supplementary Fig. 5). We also verified RML scrapie infection of CD1 coronal whole brain slice cultures (Fig. 1d), which comprised the cortex, hippocampus, thalamus, hypothalamus and midbrain (sectioning was stopped before the striatum was reached). We observed productive accumulation of prion seeding activity in CD1 whole brain slice cultures over 98 days (Fig. 1e and Supplementary Fig. 6), with proteinase K resistant PrP^Sc^ (PrP^RES^) detectable at 84 days post infection (Fig. 1f and Supplementary Fig. 7). Collectively, these findings confirmed that the cerebellar and whole brain slice cultures used in this study were of sufficient quality support prion replication.

### RML scrapie productively infects deer mouse organotypic brain slice cultures

To evaluate the potential of deer mouse slice cultures to support prion replication, we compared prion replication dynamics in CD1 and deer mouse organotypic brain slice cultures over an extended culture period of 168 days. This duration mirrors the typical incubation periods of wildtype mice infected with prions in vivo, making it a rigorous test for detecting productive prion infection in POSCA.

**Fig. 2:**
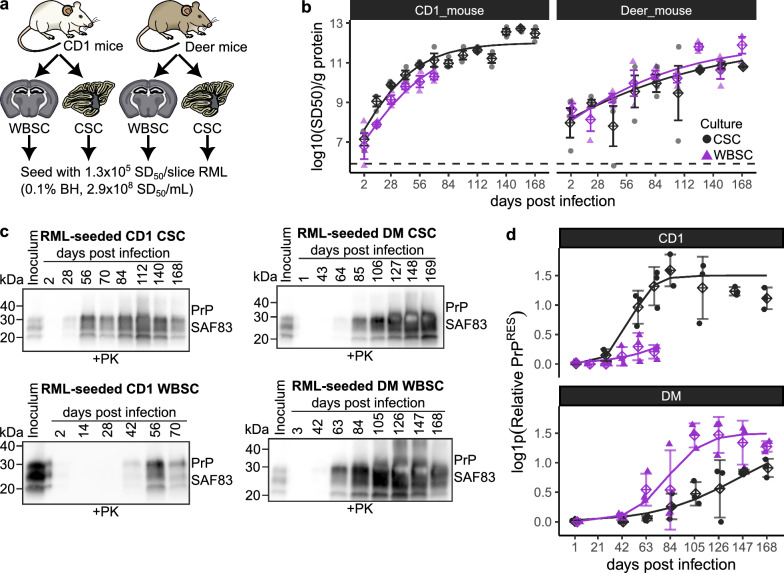
Long-term RML scrapie infection in CD1 and deer mouse cerebellar and whole brain slice cultures. **a** Cerebellar and whole brain slice cultures from both CD1 and deer mice were seeded with RML scrapie at a dose of 1.3 × 10^5^ SD_50_ units per slice. **b** Prion replication curves were produced by monitoring PrP^Sc^ seeding activity over an extended period, up to 168 days post-infection (dpi), using RT-QuIC. **c–d** Accumulation of proteinase K-resistant PrP^RES^ was measured in infected slice cultures via western blotting, showing differences in prion accumulation across species and brain regions over time. Curves in **b** and **c** represent amyloid seeding activities and PrP^RES^ measurements fitted to a sigmoidal logistic growth model, with statistical parameters (plateau time and exponential rate *k*) provided in statistical source data 1

We inoculated both deer mouse and CD1 cerebellar and whole brain slice cultures with RML scrapie (Fig. 2a). By measuring prion seeding activity every 14–21 days over the 168-day study period, we generated prion replication curves for each of the four experimental conditions (Fig. 2b and Supplementary Fig. 8).

Additionally, we compared the accumulation of proteinase K resistant PrP^Sc^ (PrP^RES^) across the four experimental paradigms (Fig. 2c and d, Supplementary Fig. 9 and Supplementary Fig. 10). In CD1-derived cultures, cerebellar slices showed more robust accumulation of seeding activity and PrP^RES^ compared to whole brain slice cultures. Interestingly, the opposite trend emerged in deer mouse cultures, with whole brain slices supporting prion replication more productively than cerebellar slices. This suggests that in addition to prion protein sequence differences, species-specific brain region microenvironments may influence prion replication dynamics in the slice culture model.

To assess whether RML replication plateaued in slice cultures, we fit prion seeding activities (Fig. 2b) and PrP^RES^ measurements (Fig. 2d) to sigmoidal logistic growth models (Statistical Source Data 1). This analysis suggested that RML PrP^Sc^ accumulation eventually plateaued at around 10^11^–10^12^ log_10_(SD_50_)/g protein at around 77 dpi in CD1 cerebellar slice cultures, and around 127 dpi in deer mouse whole brain slices. This ‘plateau’ phenomenon is also observed in vivo [38], underscoring the physiological relevance of the POSCA model. Interestingly, we observed an apparent ‘inflection’ in prion seeding activity in RML infected CD1 cerebellar slice cultures at around 140 dpi, when the levels of PrP^RES^ appeared to be slightly decreasing. It is possible that degradation of PrP^Sc^ filaments after prion accumulation has plateaued could lead to an increase of apparent seeding activity, and so it is beneficial to use both prion seeding activities and PrP^RES^ when tracking prion accumulation in POSCA. We did not observe a plateau in RML infected deer mouse cerebellar slice cultures, likely because RML accumulation was less productive in deer mouse slices compared to CD1 slices. We could not robustly examine whether RML prion accumulation plateaued in CD1 whole brain slices because data was not collected beyond 70 dpi for this experiment.

### Neuronal and synaptic degeneration are detected after prion seeding has plateaued in cerebellar slice cultures

To monitor slice culture viability during prion infection experiments, we routinely performed microscopy. Notably, one batch of RML infected CD1 whole brain slice cultures (Fig. 2a) appeared necrotic and non-viable at 70 days post infection, leading us to terminate the experiment at this time. We believe that this decline in viability was related to poor initial quality of this particular slice culture batch, rather than prion-induced toxicity. Nevertheless, these slices continued to show productive accumulation of prion seeding activity throughout the 70-day period (Fig. 2b and Supplementary Fig. 8). By contrast, all other groups remained viable over the full 168-day experimental timeframe, showing surprisingly minimal cytopathic effect or morphological disruptions by the final timepoint.

**Fig. 3:**
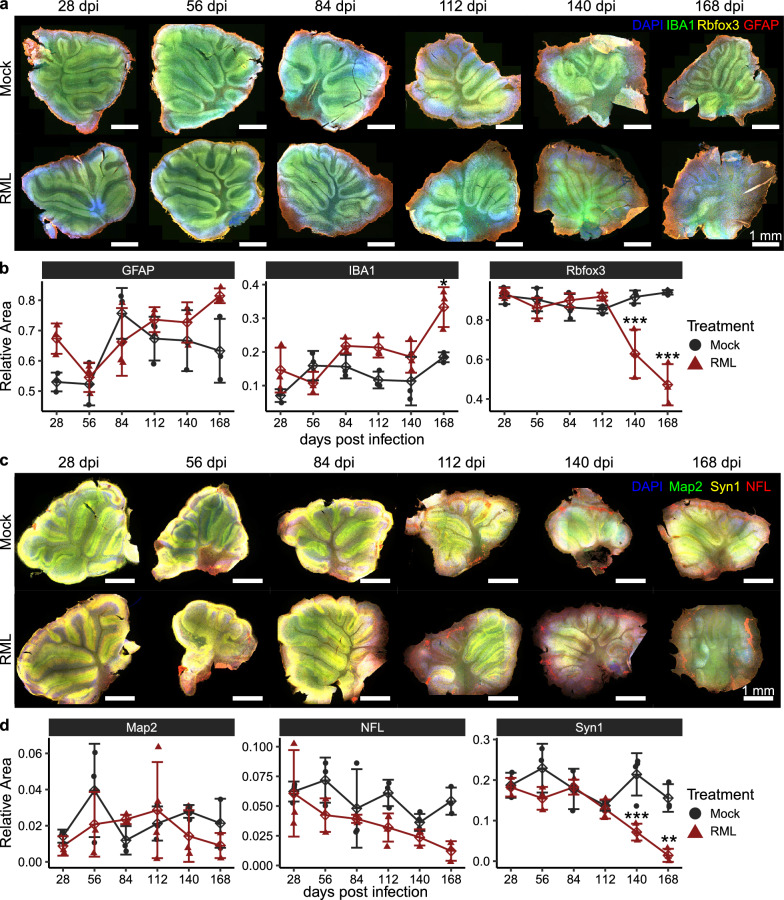
Assessment of neurotoxicity in RML-infected CD1 cerebellar slice cultures. CD1 cerebellar slice cultures were seeded with 1.3 × 10^5^ SD_50_ units of RML scrapie per slice or mock-infected with an equivalent amount of non-infectious brain homogenate. At 28-day intervals up to 168 days post-infection (dpi), fixed cerebellar sections were analyzed using immunofluorescence staining to assess neurotoxicity. **a** Immunofluorescence panels targeting Gfap (astrocytes), Iba1 (microglia), and Rbfox3 (neurons) were used to evaluate glial and neuronal cell populations. **b** Quantification of the area occupied by each marker in the regions of interest (ROIs) was performed to monitor changes in glial and neuronal markers over time. **c** Additional immunofluorescence staining for Map2 (dendrites), Nfl (neurofilaments), and Syn1 (synapses) was performed to evaluate synaptic and neuronal integrity. **d** Neurotoxicity was tracked by quantifying the area occupied by each marker in ROIs, comparing infected and mock-infected cultures. Statistical significance was determined by one-way ANOVA (*p < 0.05, **p < 0.01, ***p < 0.001)

To further investigate prion-induced cellular and molecular changes, a separate batch of CD1 cerebellar slice cultures was inoculated with RML scrapie, or Mock-infected, and tissues were fixed at 28-day intervals up to 168 days. We then performed multicolor immunofluorescence microscopy using antibodies against markers of prion neuropathology (Iba1, Rbfox3, and Gfap, Fig. 3a and Supplementary Fig. 11) and synaptic integrity (Map2, Nfl, and Syn1, Fig. 3c and Supplementary Fig. 12). For each slice, three regions of interest (ROI’s) were selected from the cerebellar granule layer, and z-stacked images of these ROIs were quantified to assess marker expression.

Comparison of prion- and mock-infected cerebellar cultures revealed limited elevation of glial markers in association with prion infection. While Gfap levels remained unaffected by prion infection, Iba1 expression increased only at the final timepoint (168 dpi, Fig. 2b). However, synaptic depletion and neuronal loss were more pronounced, with significant reductions in Syn1 and Rbfox3 signals detected from 140 days post infection onward (Fig. 2b). The detection of decreased Rbfox3 and Syn1 at 140 dpi closely matches the timeframe that neuronal loss is first detected in RML-infected wildtype mice in vivo [39]. However, these findings differ from typical in vivo mouse models of prion disease, where reactive glia are often more detectable than neuronal loss. This discrepancy may be due to the process of tissue sectioning triggering proliferation of glia and/or a higher baseline level of reactive glial phenotypes in slice cultures than intact brains, which could mask prion-elicited gliosis.

We detected neuronal loss 70 days after RML prion accumulation had plateaued in CD1 cerebellar slice cultures. Based on the observed replication of RML in deer mouse cerebellar slice cultures, we would not expect to observe evidence of neuronal degeneration, and so we restricted this analysis to CD1 tissues. Nevertheless, these results demonstrate that prion-infected wildtype slice cultures effectively model prion-induced neuronal degeneration once prion replication plateaus, faithfully recapitulating disease processes observed in vivo [39].

### Deer mouse whole brain, but not cerebellar, slice cultures replicate mouse-adapted sCJD prions

**Fig. 4:**
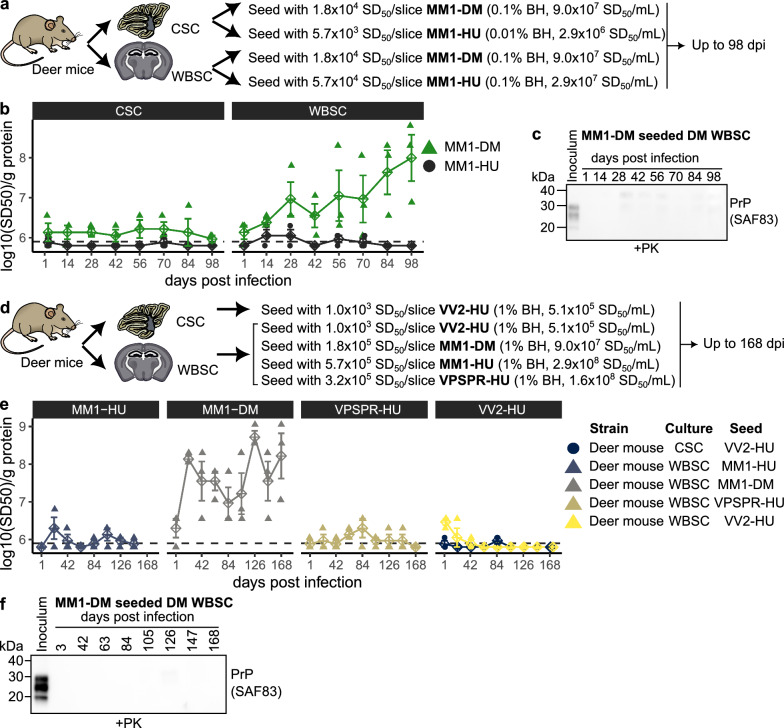
Replication of mouse-adapted sCJD MM1 prions in deer mouse whole brain slice cultures. **a** Deer mouse cerebellar (CSC) and whole brain slice cultures (WBSC) were prepared and seeded with either human (MM1-HU) or mouse (MM1-DM, third passage) sCJD-MM1 brain homogenates. **b** PrP^Sc^ seeding activity was monitored bi-weekly for 98 days post-inoculation using RT-QuIC. **c** Proteinase K-resistant PrP^Sc^ was not detected after 98 days in whole brain slice cultures seeded with deer mouse-passaged sCJD MM1 prions. **d** Additional infections were performed with deer mouse cerebellar slice cultures seeded with human sCJD VV2 (VV2-HU) and deer mouse whole brain slice cultures seeded with various prion strains: human sCJD VV2, rodent-adapted sCJD MM1, human sCJD MM1, and human VPSPR (VPSPR-HU). **e** PrP^Sc^ seeding activity in each group was tracked over 168 days post-inoculation with RT-QuIC. **f** Proteinase K-resistant PrP^Sc^ was not detected after 168 days in whole brain slice cultures seeded with deer mouse-passaged sCJD MM1 prions

To determine if deer mouse slice cultures support replication of human prions, we tested their response to inoculation with sCJD prions. Previous studies by our group have shown deer mice to be highly susceptible to sCJD MM1 prion infection in vivo, with a short incubation period of 169 days (± 10.4) after three passages (J. Myskiw et al., manuscript in preparation), comparable to the incubation period of RML scrapie in CD1 mice (136 ± 3.1 days). We challenged both cerebellar and whole brain slice cultures from deer mice with deer mouse-adapted sCJD MM1 (passaged 3 times, MM1-DM) and human sCJD MM1 (MM1-HU) inocula (Fig. 4a).

In an initial series of experiments, prion replication was assessed via RT-QuIC-based measurements of PrP^Sc^ amyloid seeding activity every 14 days up to 98-days post infection (Fig. 4b and Supplementary Fig. 13). Encouragingly, deer mouse-adapted sCJD MM1 PrP^Sc^ replicated approximately 100-fold in deer mouse whole brain slice cultures by day 98 (Fig. 4b). However, proteinase K resistant PrP^Sc^ was undetectable, even after PTA precipitation (Fig. 4c and Supplementary Fig. 14), likely due to the relatively low amyloid seeding levels by the end of the 98-day period. Based on the matching prion seeding and PrP^RES^ measurements in Fig. 2, our western blot methodology appears to detect PrP^RES^ only when seeding activity exceeds 9 log_10_(SD_50_)/g protein. In contrast to whole brain slices, cerebellar slice cultures showed minimal PrP^Sc^ seeding activity, with no increase across the 98-day time course, suggesting it was residual inoculum-derived PrP^Sc^ (Fig. 4b). Similarly, a human brain homogenate from a sCJD MM1 case did not replicate in either cerebellar or whole brain slice cultures, which showed negligible seeding activity (Fig. 4b). We also observed no prion replication in CD1 cerebellar slices after 98 days post inoculation with the mouse and human MM1 homogenates, validating them as a negative control for residual inoculum-derived PrP^Sc^ (Supplementary Fig. 15). These findings confirm brain-region-specific prion replication in organotypic cultures, with whole brain slices better suited to support sCJD prion replication than cerebellar slices.

Next, we conducted an additional series of experiments to rigorously test deer mouse slice cultures’ suitability for human prion infection over 168 days. We seeded deer mouse whole brain slices with a higher dose (1% brain homogenate) of the same mouse and human sCJD-MM1 homogenates, as well as human homogenates from sCJD-VV2 (VV2-HU), and VPSPR (VPSPR-HU) cases (Fig. 4d). Since cerebellar pathology is linked to sCJD-VV2, we also tested the VV2-HU inoculum on cerebellar slices. Prion seeding activity was monitored every 21 days over 168 days (Fig. 4e and Supplementary Fig. 16). As expected, prion seeding activity accumulated in whole brain slices challenged with rodent-adapted sCJD-MM1, reinforcing our earlier observations. However, we again failed to detect proteinase K resistant PrP^Sc^ in these rodent-adapted sCJD-MM1 seeded whole brain slice cultures (Fig. 4f and Supplementary Fig. 18), suggesting inefficient prion replication. Therefore, we would not expect to observe evidence of toxicity in these MM1-DM infected deer mouse whole brain slice cultures. Furthermore, none of the human inocula – despite high dose and extended incubation – produced detectable infection in deer mouse brain slices, underscoring a resistance to human prions in vitro that contrasts with the susceptibility observed in vivo.

### Kinetic analysis of prion propagation reveals replication rate differences across species, cultured brain regions, and prion strains

**Fig. 5:**
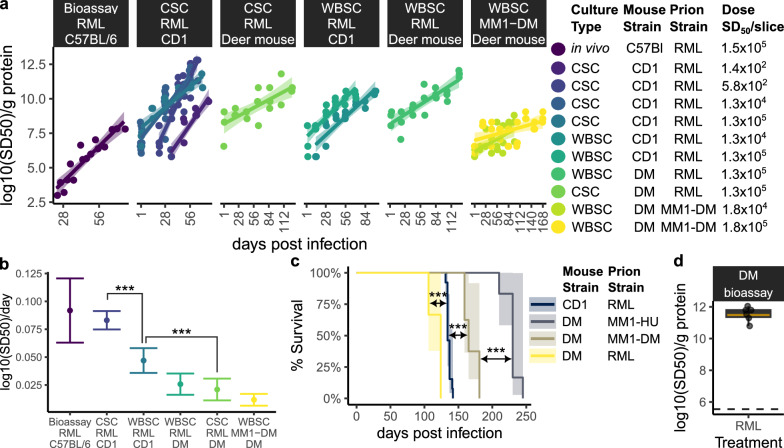
Variation in prion replication rates across prion strains, brain regions, and experimental paradigms. **a** Prion replication rates were assessed in cerebellar (CSC) and whole brain slice cultures (WBSC) infected with RML scrapie and deer mouse-passaged sCJD MM1 (MM1-DM) by plotting log_10_(SD_50_) against days post-infection (dpi) and applying linear regression. Only samples collected during the exponential phase of prion replication were included for rate analysis. To calculate a single replication rate per experimental condition, Δdpi was computed for each inoculum dosage tested and all data points per condition were fitted to a single linear regression model. Replication rates of RML prions in vivo were also analyzed by fitting log_10_(infectivity) over dpi using previously published wild-type mouse bioassay data from Meisl et al. [40]. **b** One-way ANOVA was used to compare prion replication rates across experimental conditions. **c** To assess differences in prion replication trends between POSCA and in vivo models, Kaplan–Meier curves were used compare the incubation periods between RML-infected deer mice with relevant prion mouse bioassays from ongoing, related studies. Statistical significance is denoted as *p < 0.05, **p < 0.01, ***p < 0.001. **d** Prion seeding activity was quantified in brain homogenates from deer mice at the clinical endpoint following RML scrapie infection

We investigated whether the timeframe of PrP^Sc^ accumulation in POSCA correlates with prion replication rate, as earlier work reported that in vitro RML scrapie replication in cerebellar slices is "quantitatively similar to amplification in vivo, but fivefold faster" [32]. As direct comparisons of in vitro and in vivo prion replication rates were not made in that study, we leveraged our own dataset for such an analysis. Prion replication rates were calculated using linear regression of log_10_(SD_50_) versus dpi for each experiment where productive prion infection was detected (Fig. 5a). Only samples in the exponential phase, showing a linear increase in log_10_(SD_50_) before plateauing, were included, resulting in different time frames across paradigms for calculating prion replication rates. Additionally, we compared in vitro rates to in vivo RML replication in mice using previously published infectivity data from Meisl et al. [40] and Sandberg et al. [38]. Prion replication rates, measured from the slopes of fitted regression lines, were compared across conditions (Fig. 5b).

The RML replication rate in CD1 cerebellar slice cultures (0.083 log_10_(SD_50_)/day; SE = 0.0042) closely matched in vivo mouse bioassays (0.092 log_10_(SD_50_)/day; SE = 0.015). RML replicated significantly faster in CD1 cerebellar slices than in CD1 whole brain slices (0.047 log_10_(SD_50_)/day; SE = 0.0056). No significant difference was observed between CD1 and deer mouse whole brain slices, with deer mouse slices replicating RML at 0.026 log_10_(SD_50_)/day (SE = 0.0049). Deer mouse cerebellar slices exhibited the slowest RML replication rate at 0.021 log_10_(SD_50_)/day (SE = 0.0049). Deer mouse-adapted sCJD MM1 in deer mouse whole brain slices also showed a notably slow replication rate of 0.012 log_10_(SD_50_)/day (SE = 0.0028).

Based on these rates, we expect a tenfold increase in RML prion seeding activity approximately every 11 days in CD1 cerebellar slices (matching in vivo bioassays), every 21 and 38 days in CD1 and deer mouse whole brain slices, respectively, and every 47 days in deer mouse cerebellar slices. Similarly, we estimate a tenfold increase in prion seeding activity every 85 days in deer mouse whole brain slices challenged with mouse-adapted sCJD MM1.

Unexpectedly, the trends in prion replication rates in POSCA differed from in vivo trends. Kaplan–Meier analysis showed that, contrary to slice cultures, RML prion disease progressed faster in deer mice than CD1 mice when inoculated intracranially in vivo (Fig. 5c). To confirm in vivo RML infection in deer mice, we measured prion seeding activities in brain homogenates, which averaged around 11.5 log_10_(SD_50_)/g protein (Fig. 5d and Supplementary Fig. 18), aligning with disease endpoints typical of murine prion infections.

This analysis of prion replication rates (Fig. 5b) indicates that brain slice cultures can, at best, replicate prions at rates comparable to in vivo models. However, replication rates vary by species, brain region, and prion strain. The notably slow replication of mouse-adapted sCJD MM1 prions relative to RML scrapie could partly explain the difficulty in developing slice culture models compatible with human prion infection.

### Cellular and molecular differences between cultured brain sections and in vivo brain tissues may influence prion replication

**Fig. 6:**
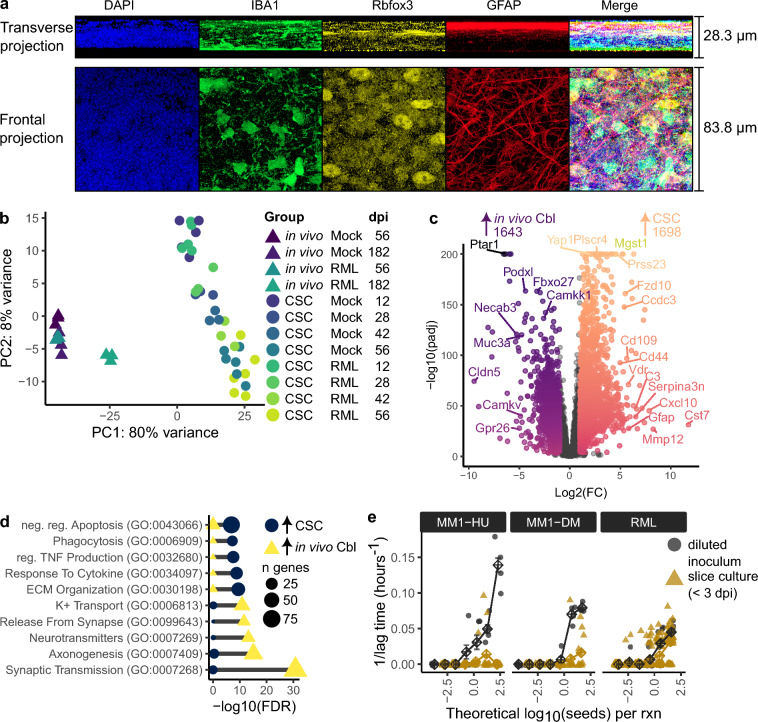
Differences in cellular and molecular composition between cultured cerebellar sections and in vivo cerebellar tissue. **a** Representative frontal and transverse projections from z-stacked fluorescence microscopy images of Mock-treated CD1 cerebellar slice cultures stained for Iba1 (microglia), Rbfox3 (neurons), and GFAP (astrocytes) at 28 days post infection (dpi). **b** Principle component analysis (PCA) plot showing variation in gene expression between in vitro and in vivo cerebellar tissues. **c** Volcano plot showing differentially abundant transcripts between cerebellar slice cultures (CSC) and in vivo cerebellar tissue (Cbl) based on RNA sequencing analysis. **d** Dot plot highlighting key biological processes enriched among differentially expressed genes in cultured versus in vivo cerebellar tissues. **e** Quantification of residual inoculum seeding activity in cerebellar slice cultures (samples collected within the first 3 dpi) challenged with MM1-HU, MM1-DM, and RML. Seeding activity was assessed by plotting inverse lag time (1/lag time) measurements in RT-QuIC against the theoretical seed count per reaction and comparing the observed residual inoculum signal to the expected inoculum signal

The inefficient replication of deer mouse-adapted sCJD MM1 prions in deer mouse slice cultures cannot be attributed to species barriers alone, suggesting that microenvironmental differences between cultured slices and in vivo brain tissue may play a role. In cerebellar slice cultures, glial and neuronal cells were organized in distinct layers, with Gfap^+^ astrocytes forming a dense network on the slice surface where nutrients from the culture media are most accessible (Fig. 6a). Rbfox3^+^ neurons were concentrated just below the astrocyte layer, where nutrients and oxygen were also likely more available. Iba1^+^ microglial cells were abundant throughout the slice.

To further investigate cellular and molecular changes within cerebellar slices during culturing, we analyzed a previously published RNA sequencing dataset made from RML-infected cerebellar slice cultures and in vivo cerebellar tissues [41], similar to the slice cultures used here. Differential gene expression analysis revealed numerous transcriptional differences between cerebellar slice cultures and in vivo cerebellar tissues (Fig. 6b–d). Using stringent criteria (FDR < 0.001 and |log2FC|> 1), we identified gene expression signatures associated with each context (Fig. 6c). Compared to in vivo tissues, cerebellar slice cultures exhibited elevated expression of genes associated with inflammatory signaling and phagocytosis, but reduced expression of genes involved in synaptic transmission and neuronal function (Fig. 6d). These changes likely reflect alterations in cellular composition following sectioning and culturing, with cerebellar slice cultures showing reactive astrocytes and microglia not typically found in healthy in vivo brain tissues (Fig. 3a and Supplementary Fig. 11).

The presence of reactive glia in slice cultures could contribute to PrP^Sc^ degradation, a hypothesis supported by previous studies where microglial depletion led to increased prion accumulation in cerebellar slices [32]. To assess prion degradation or clearance within slice cultures, we estimated residual inoculum seeding activities in slice cultures at the first timepoint collected for RT-QuIC analysis (< 3 dpi). To estimate the observed residual inoculum signal, we plotted RT-QuIC lag^−1^ measurements against the theoretical number of prion seeds per reaction, calculated from the known seeding titers of the inocula used (Fig. 6e and Supplementary Fig. 19). This was compared with the expected inoculum signal assuming no PrP^Sc^ degradation, which was estimated by plotting the corresponding inoculum lag^−1^ measurements on the same graph. Although RML was not particularly sensitive to degradation in our study, we observed lower-than-expected baseline residual inoculum seeding activity in deer mouse slices challenged with human sCJD MM1 prions (Fig. 6e). This could suggest that human sCJD MM1 prions are especially sensitive to degradation or clearance within slice cultures, potentially contributing to the differential susceptibility of deer mouse tissues to sCJD prions in vitro versus in vivo.

Additionally, we observed that many molecular changes in cerebellar slice cultures involved extracellular matrix (ECM) composition (Fig. 6d). Prior studies have linked ECM gene expression with susceptibility to prion propagation [42], suggesting that these ECM-related gene expression changes could influence the susceptibility of slice cultures to various prion strains.

## Discussion

It is often challenging to interpret how well cell culture models reproduce prion infection in relation to animal bioassays—the gold standard prion model. Here, we demonstrated that while RML-seeded CD1 cerebellar slice cultures closely matched animal bioassays, deer mouse slice cultures were not equivalent to in vivo models. This finding is supported by our kinetic analysis of prion propagation in cultured brain slices, which revealed that prion replication rates vary based on the mouse species and brain region used, irrespectively of PrP sequence homology. We also highlighted cellular and molecular differences between cerebellar slice cultures and in vivo tissues, and demonstrated differences in replication rates and initial inoculum signals between sCJD MM1 and RML prion strains. Collectively, these findings clarify POSCA’s utility for prion disease research and imply a relationship between the molecular environment and the susceptibility of cultured brain slices to prion infection.

Our findings demonstrate POSCA’s potential for comparing prion replication rates, which differed between species, brain regions, and prion strains. Such comparisons could enhance reproducibility and provide deeper insights into strain-specific replication dynamics. For instance, POSCA’s physiological faithfulness [34] was demonstrated by RML scrapie’s prion replication rate that closely matched between cerebellar slice cultures and in vivo mouse bioassay [38, 40], even eliciting neurotoxicity at similar timepoints [39]. Specifically, we calculated an exponential rate of 0.091 log_10_(SD_50_)/day for RML prions in CD1 cerebellar slices. This aligns with an earlier study that reported prion growth rates of 0.05–0.17 log_10_(infectivity)/day across various animal models and prion strains, including RML, 139A, 263 K, sc237, Fukoka-1 hamster-adapted CJD, and mouse-adapted CJD [43]. Mouse-adapted CJD prions were found to replicate at approximately half the rate of RML scrapie in vivo [43], a trend we reproduced in deer mouse whole brain slice cultures. These slower kinetics of CJD prion replication may partly explain the difficulty of infecting cell cultures with human prions, particularly in dividing cellular paradigms where prion replication must outpace cell division [44].

We also observed notable differences in prion replication rates between POSCA and in vivo models that were not attributable to PrP sequence homology. In CD1 tissues, RML prions replicated more effectively in cerebellar cultures, whereas deer mouse whole brain slices better supported RML propagation. The rapid replication of RML in CD1 cerebellar slice cultures contrasts with in vivo models, where the cerebellum is not a primary site for RML replication in wildtype mice [39]. We note that here we relied on a combination of RT-QuIC and western blotting for tracking prion accumulation. Incorporation of in situ detection of PrP^Sc^ as an additional means of tracking prion replication in future studies may provide additional insight into regional differences in prion propagation. Additionally, RML replicated faster in CD1 brain slices than in deer mouse slices, despite deer mice having shorter incubation periods for RML scrapie in vivo. These discrepancies suggest that factors beyond PrP sequence homology, such as microenvironmental differences between cultured brain slices and their in vivo counterparts, may influence prion replication dynamics.

The resistance of deer mouse slice cultures to infection with human sCJD MM1 prions is intriguing, given their susceptibility to the same inoculum in vivo. Deer mice developed disease after 229 days (± 11) on first passage of MM1-HU, and the MM1-DM inoculum (third passage from the same human inoculum) was associated with an incubation period of 169 days (± 10.4) (J. Myskiw et al., manuscript in preparation). Even MM1-DM replicated poorly in deer mouse slice cultures, and did not produce detectable proteinase K resistant PrP^Sc^ after 168 days. This discrepancy indicates suboptimal replication of sCJD MM1 prions in slice cultures, even after extensive in vivo adaption. We cannot attribute this resistance to a species barrier, given the matching PrP sequence between inoculum and slice cultures, again pointing to an influence from tissue microenvironment.

There are several potential explanations for the resistance of deer mouse slice cultures to sCJD prion infection that warrant discussion. For instance, we speculate that MM1-DM and MM1-HU prions might be cleared from slice cultures based on the unexpectedly low residual inoculum signal observed for MM1-DM and MM1-HU. The observation of potential inoculum clearance in slice cultures contrasts with other in vitro [45] and in vivo [46] paradigms where inoculum-derived human PrP^Sc^ persists indefinitely. However, our estimation of inoculum clearance could have been influenced by interactions between PrP^C^ in the slice culture and PrP^Sc^ in the inoculum. Additional experiments using *PRNP*^*−/−*^ mice and additional human isolates are warranted to further assess inoculum degradation and clearance in the slice culture model. Additionally, the slow accumulation of prion seeding activity in MM1-DM-seeded slice cultures hints that further in vivo adaption might enhance prion propagation in slice cultures. Prions extensively passaged in mice, like RML scrapie, may be better suited to slice cultures due to selection for infectious, degradation-resistant, or faster-replicating PrP^Sc^ species. Human sCJD prions, in contrast, have not undergone such selection, which could contribute to the inherent difficulty of initiating prion replication in cell cultures using human PrP^Sc^. Our study, however, only compared third passage MM1-DM with MM1-HU, and so further experiments that comprehensively examine prion replication in slice cultures after sequential passages in deer mice for multiple prion strains would shed further light on this question. Thus, while we cannot fully explain the differential prion susceptibilities between deer mouse slice cultures and animal bioassays, our findings put forth several areas for further investigation.

## Conclusions

The major conclusion from this study was that efficient prion replication in vivo is not necessarily predictive of successful propagation in slice cultures. This is based on our observation of limited replication of MM1-DM prions in deer mouse slice cultures despite rapid disease progression in intracerebrally inoculated mice. The disconnect between PrP sequence homology and prion susceptibility of cultured brain slices implies an influence from tissue microenvironment, consistent with the theory that efficient prion replication requires a molecular environment enriched with certain cofactors [47–50]. The idea that PrP alone is insufficient for infection is supported by our previous inability to infect human PrP^C^-overexpressing neural progenitor-derived cultures with human prions [45]. Furthermore, our finding of prion-strain-dependent differences in replication rate and estimated residual inoculum signal in slice cultures is also consistent with evidence indicating that optimal cofactor composition may vary between prion strains [47, 51]. As our knowledge of the molecular factors driving prion replication improves, it may become possible to develop more rational strategies for effectively infecting cell cultures with human PrP^Sc^.

## Materials and methods

### Mice

All procedures that involved live animals conformed to the ethical guidelines of the Canadian Council on Animal Care (CCAC) and were approved under animal use document (AUD) H15-032 or AUD H20-001 by the Animal Care Committee (ACC) of the Canadian Science Centre for Human and Animal Health (CSHAH). Timed-pregnant CD1 mice were purchased from the University of Manitoba and transported to the animal holding facilities at the Canadian Science Centre for Human and Animal Health (CSCHAH) on embryonic day 17 (E17). The resulting pups were kept together with the mother until postnatal day 9–12 (P9-P12), at which point they were euthanized via isoflurane anesthesia and cervical dislocation. Deer mouse pups were transported from the University of Manitoba on postnatal day 9–12 and immediately euthanized upon arrival at the CSCHAH animal facility (timed-pregnant deer mice were not used in order to minimize any impact to breeding at the U of M colony). Fresh brain tissues were collected from the P9-P12 pups and used immediately for the production of neural organotypic slice cultures.

### Preparation of organotypic cerebellar and whole brain slice cultures

Cerebellar sections were produced as described previously [33, 52]. Briefly, the cerebella were dissected and collected in ice-cold GBSS (Geys balanced salt solution; 137 mM NaCl, 5 mM KCl, 0.845 mM Na_2_HPO_4_, 1.5 mM CaCl_2_*2H_2_O, 0.66 mM KH_2_PO_4_, 0.28 mM MgSO_4_*7H_2_O, 1.0 mM MgCl_2_*6H_2_O and 2.7 mM NaHCO_3_; pH 7.2–7.4) supplemented with 1 mM Kynurenic acid and 0.6% glucose (GBSSK). Meninges were removed and cerebella were submerged in ultra-low melting-point agarose, which was chilled on ice until it solidified, cut to form a cube around the cerebellum, and glued to a vibratome mounting disc. The mounting discs were submerged in the vibratome buffer chamber with GBSS that was kept at 4 °C with a cooling block. The Leica VT1200S vibratome was used to cut 300 μm thick sagittal cerebellar sections which were released from the agarose using Dumont #5 forceps before collecting in ice-cold GBSSK. A blunted Pasteur pipette was used to carefully transfer the cerebellar sections to 6-well plates that contained Millicell-CM transwell inserts with ice-cold GBSSK. Excess liquid was removed from the apical surface of the transwell inserts. Once all the slices were plated, the transwell inserts were transferred to a new 6-well plate that contained 1 mL of slice culture medium (SCM) in each well. SCM was based on the recipe reported by Opitz-Araya, and consisted of MEM eagle medium supplemented with 20% horse serum, 1 mg/L insulin, 5.2 mM NaHCO_3_, 30 mM HEPES, and 1× Gibco Antibiotic–Antimycotic solution (0.1 mg/mL penicillin, 0.1 mg/mL streptomycin, and 250 ng/mL Amphotericin B) [53]. The cultured cerebellar sections were maintained in a tissue culture incubator at 37 °C and 5% CO2 with the complete volume (1 mL) of SCM being exchanged three times per week.

Whole brain slice cultures were produced according to the same protocol with minor adjustments. The cerebrum was dissected and cut along the posterior-anterior axis to produce 2 halves that were embedded in agarose for sectioning. Whole cerebral brain tissues were sectioned coronally at 200 μm, beginning at the level of the midbrain and stopping before the level of the striatum. The resulting sections were too big to transfer with a blunted Pasteur pipette, and so they were deposited on the membrane culture insert immediately after they were produced.

### Prion infection of organotypic brain slice cultures

Brain slices were inoculated with prions either pre- or post-culturing. According to the original pre-culture inoculation protocol [33], groups of 10 cerebellar sections were incubated in 1 mL of brain homogenate diluted in GBSSK for 1 h at 4 °C in the fridge while shaking. Following incubation with inoculum, the cerebellar sections were washed 3 times with 5 mL of GBSSK before transferring to transwell inserts and culturing. Alternatively, a small volume of inoculum (2 μL diluted in PBS) was directly applied onto each cultured brain slice (cerebellar or whole brain) after 1 day in vitro (div). Following inoculation, the slices were maintained in culture by exchanging the complete volume of SCM three times per week.

The source of inocula were 10% brain homogenates, which were prepared in PBS by two 30 s sonication bursts followed by two 10 min centrifugations at 2,000 *xg*. Inoculum was prepared from CD1 mouse brains collected at the clinical endpoint following inoculation with Rocky Mountain Laboratory scrapie (RML) or non-infectious brain homogenate (Mock). Inoculum was also made from deer mouse brain tissue collected at the endpoint of the third passage of infection with deer-mouse adapted sCJD MM1 (MM1-DM). Human inoculum was prepared from cortical brain tissues collected post-mortem from individuals with sCJD MM1 (MM1-HU), sCJD VV2 (VV2-HU) and variably protease-sensitive prionopathy (VPSPR-HU). Human tissues were subtyped by the Canadian CJD Surveillance System (CJDSS) through standard diagnostic genotyping and PrP^Sc^ glycotyping and approved for use in research with appropriate consent under Health Canada-Public Health Agency of Canada (HC-PHAC) Research Ethics Board (REB) reference number REB 2017-009P. PrP^Sc^ amyloid seeding activity was characterized in the different inocula batches (Supplementary Fig. 20) and this was used to calculate the dosage of inoculum-derived PrP^Sc^ seeding activity used in each experiment.

In one experiment, RML inoculum was spiked with nanoparticles prior to being applied to slice cultures. Nanocomposix 100 nm diameter Silica Nanospheres (Si), or 100 nm diameter Mesoporous Silica Nanospheres (Me) were diluted in RML inoculum to final concentrations of 25, 2.5, or 0.25 µg/mL.

### Proteinase-K (PK) digestion and western blotting

Cerebellar and whole brain slice cultures were collected and homogenized in 100 µL of PBS via two 30 s sonication bursts and protein content was determined using the Pierce BCA assay. For specific detection of PrP^Sc^, 20 μg of lysate was digested with 25 μg/mL of proteinase K (PK) in a 20 μL reaction (in PBS with 0.5% Na-deoxycholate and 0.5% NP-40) at 37 °C for 30 min. In one experiment, slice culture lysate was precipitated with phosphotungstate anion (PTA) prior to PK digestion. Briefly, 100 μg of lysate was diluted in 250 µL of PBS with 0.5% Na-deoxycholate, 0.5% NP-40, and 2% Sarkosyl, digested with 7.5 units of Benzonase for 2 h at 37 °C, and PrP^Sc^ was then precipitated in the presence of 2% Na-PTA overnight at 37 °C. PTA-precipitated PrP^Sc^ was then pelleted by centrifuging at 16,000 *xg* for 1 h at room temperature. The entire pellet was digested with PK as above.

Lysate (either digested or not digested with PK) was diluted in laemmli buffer (BioRad) with 10 mM dithiothreitol (DTT) (Millipore-Sigma) and boiled for 5 min before electrophoresis was run on 8–16% TGX Stain-free SDS-PAGE gels (BioRad) and at 200 V. Total protein was imaged on a BioRad Gel dock imager according to the Stain Free protocol prior to transfer to nitrocellulose or low-fluorescence PVDF membranes using the BioRad Transblot system. Membranes were blocked with 5% skim milk diluted in Tris buffered saline with 0.1% Tween-20 (TBST) for 1 h at room temperature before incubating with primary antibodies at 4 °C overnight. The membranes were then washed three times for 5 min each before incubating with secondary antibodies at room temperature for 1 h. The membranes were washed four times for 5 min each and visualized using the Pierce femto-sensitivity ECL substrate kit (ThermoFisher), or via fluorescence depending on the secondary antibodies used. Densitometric quantification of western blot signal intensity was performed with imageJ. Uncropped western blot images are shown in Supplementary Figs. 5, 7, 9, 10, 14, and 17.

Antibodies: 6H4-mouse-anti-PrP (1:2000, ThermoFisher 7500996), SAF83-mouse-anti-PrP (1:5000, Caymen Chemical 189765), rabbit-anti-Gfap (1:100,000, DAKO Z0334), mouse-anti-Tubb3 (1:5000, Abcam ab78078), rabbit-anti-Rbfox3 (1:2500, Abcam ab177487), rabbit-anti-Calb1 (1:2000, Abcam ab255691), rabbit-anti-Syn1 (1:2500, Abcam ab64581), goat-anti-mouse-IRDye-800 (1:5000, LI-COR 925-32210), goat-anti-rabbit-IRDye-680 (1:10,000, LI-COR 925-68021), goat-anti-mouse-HRP (1:10,000, DAKO P0447) and goat-anti-rabbit-HRP (1:10,000, DAKO P0448).

### Real-time quaking-induced conversion (RT-QuIC)

The detection of PrP^Sc^ seeding activity by RT-QuIC was based on previously published methods [7, 45]. Slice culture lysate was serially diluted tenfold in PBS supplemented with 0.04% SDS and 0.4% N2 supplement (Gibco). RT-QuIC reactions were prepared in 96-well plates by mixing 5 μL of diluted lysate with 95 μL of a master mix to give reactions with 300 mM NaCl, 0.01 mM EDTA, 10 nM ThT, 0.002% SDS and 10 μg substrate PrP in PBS. Substrate PrP was prepared in-house via recombinant expression in bacteria followed by purification with Nickel affinity chromatography. Full-length hamster (23–231) and truncated hamster (90–231) PrP were used as substrate PrP for RT-QuIC. RT-QuIC was run on each sample dilution in quadruplicate and brain homogenate from a 263 K and mock-infected hamster was run as positive and negative technical controls. The 96-well plates were sealed with optical adhesive film and run on FLUOstar Omega microplate readers (BMG) in 15 min cycles of double-orbital shaking followed by ThT fluorescence measurements (450 nm excitation and 480 nm emission). RT-QuIC reactions were run for a maximum of 40 and 20 h when full-length and truncated hamster PrP were used substrate respectively.

Fluorescence data was processed with MARS data analysis software (BMG) and exported in csv format for further analysis using an in-house developed R package (https://github.com/jslota/rtquicR). Briefly, raw ThT fluorescence measurements was normalized per-well to the average of baseline signal collected between 2 and 4 h of the reaction, prior to amplification of amyloid filaments. Positive reactions were defined as those that exceeded a threshold fluorescence level by then end of the reaction. Threshold values were calculated per-plate as the mean + 10 standard deviations of the baseline signal collected between 2 and 4 h of the reaction. Inverse lag time values (1/lag time) were defined as the inverse of the time to reach the threshold fluorescence value. Log_10_(SD_50_) measurements were calculated using the Spearman-Karber transformation. RT-QuIC fluorescence, inverse lag time, and SD_50_ measurements are shown in Supplementary Figs. 3, 4, 6, 8, 13, 15, 16, 18, 19, and 20.

### Immunofluorescence

Cerebellar slice cultures were fixed with 4% PFA for 1 h at room temperature and washed three times with PBS. Tissues were then permeabilized with 0.5% Triton-X 100 in PBS for overnight at 4 °C and blocked with 10% goat serum + 1% BSA in PBST for 24–48 h at 4 °C. Primary antibodies were diluted in blocking buffer and incubated with the tissues for 48–72 h at 4 °C. Tissues were washed four times for 5 min with PBS with 0.1% Tween-20 (PBST) and then were incubated with the secondary antibodies in blocking buffer for 48–72 h at 4 °C. Tissues were washed six times for 5 min in PBST and then coverslips were mounted using Prolong Glass with NucBlue.

Images were acquired with a Zeiss LSM980 confocal microscope. Overview images of entire slices were acquired via tiling in widefield mode, and were used for navigation to select targeted region of interest (ROI) images of the cerebellar granule layer. We chose the granule layer because it represents a dense population of Rbfox3^+^ neurons that could be consistently targeted in different cerebellar slices. Z-stacked images of ROIs were acquired in airyscan mode. Orthogonal maximum intensity projections of ROI images were segmented separately for each channel using cellprofiler, and the area occupied by positive signal was carried forward for analysis of protein expression. For protein analysis, three ROI images were analyzed per individual slice, and three slices were analyzed per group. Protein expression measurements were reported as the average area occupied across the three ROI images per slice. Representative z-projections of ROI images are shown in Supplementary Figs. 11 and 12.

Antibodies: rabbit-anti-Calb1 (1:100, Abcam ab255691), chicken-anti-Nfl (1:500, Abcam ab24520), mouse-anti-Map2 (1:500, Abcam ab11267), rabbit-anti-Syn1 (1:500, Abcam ab254349), rabbit-anti-Rbfox3 (1:500, ThermoFisher 702022), chicken-anti-Gfap (1:500, Abcam ab4674), rat-anti-Iba1 (1:2,000, Abcam ab283346), goat-anti-mouse-Alexa488 (1:1000, ThermoFisher A11001), goat-anti-rat-Alexa488 (1:1000, ThermoFisher A21212), goat-anti-rabbit-Alexa555 (1:1000, ThermoFisher A32732), goat-anti-chicken-Alexa647 (1:1000, ThermoFisher A32933), goat-anti-rabbit-Alexa647 (1:1000, ThermoFisher A21245).

### Analysis of previously published RNAseq data

The RNA sequencing dataset of in vitro and in vivo cerebellar tissues previously published by Liu et al. [41] was retrieved from ArrayExpress under accession numbers E-MTAB-11635 and E-MTAB-11742. Raw sequencing reads in fastq format were pre-processed according to our previously described pipeline [54] by cleaning reads with Trimmomatic, removing rRNA reads with Bowtie2, mapping reads to the mouse genome (mm10) with HISAT2, and counting transcripts with FeatureCounts. DESeq was used for normalization, principle component analysis, and differential expression analysis, fitting tissue origin (cerebellar slice cultures or in vivo cerebellar tissues), RML disease status, antibody treatment and timepoint as co-variates in DESeq2s negative binomial regression model. Criteria of FDR-corrected *p* value < 0.001 and |log2FC|> 1 were used to identify transcripts that significantly differed between in vitro and in vivo cerebellar tissues across the entire dataset irrespective of the other variables. Gene-ontology (GO) biological processes enriched with differentially expressed transcripts were identified with Enrichr.

### Statistics

Protein expression levels were compared between treatment groups using one-way analysis of variance (ANOVA), followed by Tukey's post-hoc tests for pairwise comparisons. Data were derived from western blot and immunofluorescence experiments. For western blot data of healthy CD1 cerebellar slice cultures over time, Imagej-derived densitometric quantifications of PrP, Calb1, Tubb3, Rbfox3, Syn1, and Gfap were normalized to total protein signal (*n* = 3 slices per timepoint). Significant changes in protein abundance between each timepoint (e.g. 30 vs. 60 div) were identified based on Tukey-adjusted *p*-values. For immunofluorescence data comparing RML- and Mock-infected CD1 cerebellar slice cultures over time, normalized areas for markers (Gfap, Iba1, Rbfox3, Syn1, Map2, and Nfl) were calculated from segmented images. Normalized values were aggregated per tissue slice (*n* = 3 images per slice) and grouped by days post-infection (dpi) and treatment for statistical comparisons (*n* = 3 slices per group). Time-matched group differences (e.g., RML at 28 dpi vs. Mock at 28 dpi) were analyzed using Tukey-adjusted *p*-values to identify significant prion-associated changes over time.

Prion replication rates were calculated by fitting log_10_(infectivity) or log_10_(SD_50_) measurements against days post infection (dpi) using linear regression models. To focus on the exponential phase of prion replication, only samples collected from first detectable signal via RT-QuIC until the plateau phase were included. Data were normalized within each group by defining the dpi relative to the earliest timepoint used in each experiment: Δdpi = dpi_n_ – dpi_0_. Linear regression was performed for each experimental groups, which was defined by combinations of medel type, species, prion strain, and inoculum dosage. Pearson correlations between log_10_(SD_50_) and Δdpi were also calculated to validate linear relationships within each group. Differences in replication rates (slopes) were compared using analysis of variance (ANOVA), followed by post hoc pairwise comparisons with Tukey’s adjustment for multiple testing. R code was used for data processing, visualization, and statistical analysis using the dplyr, ggplot2, and lsmeans packages respectively.

To model the plateau effect in prion replication data, log_10_(SD_50_) measurements or log1p(PrP^RES^) measurements were fit to the following sigmoidal logistic growth model: $P\left(t\right)=\frac{Pmax}{1+{e}^{-k(t-{t}_{1/2})}}$, where *P(t)* is the amount of PrP^Sc^ at time *t*, *Pmax* is the maximum concentration of PrP^Sc^ at the plateau phase, *k* is the exponential rate constant, and *t*_*1/2*_ is the inflection timepoint at which PrP^Sc^ reaches half of *Pmax*. The timepoint at which the plateau phase was achieved was defined as the point at which PrP^Sc^ reaches 0.95 * *Pmax*, and was calculated from *k* and *t*_*1/2*_: $plateau time= {t}_{1/2}+\frac{\text{log}(0.95/0.05)}{k}$.

Kaplan–Meier survival analysis was performed to compare the in vivo incubation periods of prion-infected animals from ongoing, related studies (J. Myskiw et al., manuscript in preparation). Live animal prion infections were approved under AUDs H-11–020 and H-19–005. CD1 mice were intracranially inoculated with RML, while deer mice were intracranially inoculated with RML, MM1-HU, and MM1-DM. Survival data were grouped by species and treatment and summarized to calculate mean incubation periods and standard errors (SE). Kaplan–Meier survival curves were generated using the survival package in R. Group differences in incubation period were assessed using the log-rank test, and pairwise comparisons were conducted via a parametric survival regression model with Tukey-adjusted post hoc tests using the multicomp package.

Results from statistical analyses are provided in Statistical Source Data 1.

## Supplementary Information

Additional file 1.

Additional file 2.

## Data Availability

The data that support the findings of this study are available from the corresponding author upon reasonable request.
