# Supplementary material for: Prion replication in organotypic brain slice cultures is distinct from in vivo inoculation and is species dependent
Source: Acta Neuropathol Commun. 2025 Apr 30;13:86. doi: 10.1186/s40478-025-01999-w (PMC12042311; doi:10.1186/s40478-025-01999-w)
Supplement: Supplementary file 1 — Additional file 1. [file 40478_2025_1999_MOESM1_ESM.docx]

**Additional details pertaining to optimizing POSCA**

**Slice thickness**

We found that 300 μm cerebellar slices were better preserved upon culturing than thicker 350-400 μm slices that are often used in POSCA (Supplementary Figure 2c). Inflammation and tissue degradation was more pronounced when the cerebellar sections were incubated in the fridge at 4 °C for 1 hr prior to culturing - the standard method for prion inoculation of slice cultures^1^. Consequently, we selected a cerebellar section thickness of 300 μm for subsequent experiments.

We also examined morphology of one batch of CD1 whole brain slice cultures made at thicknesses ranging from 150 to 300-µm (Supplementary Figure 2h). We selected a whole brain slice thickness of 200 µm because these slice cultures exhibited better preservation than 250-300 µm slices.

**Protein expression in healthy CD1 cerebellar slice cultures**

To verify slice culture longevity, we evaluated the survival of neural cell populations based on protein biomarker expression in healthy CD1 cerebellar slice cultures over 90 days *in vitro*. Western blotting confirmed the expression of neuronal and glial proteins over 90 days *in vitro* (div) (Supplementary Figure 2d and 2e). Importantly, the sustained expression of PrP^C^ indicated that these cerebellar slice cultures were suitable for replicating PrP^Sc^. The mature neuron marker Rbfox3 and synaptic marker Syn1 were sustained even at 90 days *in vitro* (div), whilst the abundance of Calb1 and Tubb3 indicated a possible depletion of Purkinje and immature neurons, respectively. Immunofluorescence staining for Calb1 confirmed the presence of viable Purkinje neurons, a difficult cell type to culture *in vitro* (e.g.^2^), after 30 and 65 days *in vitro* (Supplementary Figure 2f and 2g). These data suggest that neuronal subsets associated with prion replication remained viable.

**Direct inoculation of slices post-culturing**

We then optimized conditions for seeding prion replication in POSCA. Typically^1^, free-floating cerebellar sections are inoculated by soaking in 1 mL of diluted prion-containing brain homogenates at 4 °C for 1 hour before culturing. Here we evaluated an alternative approach, applying a small volume (2 µL) of prion inoculum directly onto cerebellar sections post-culturing (Figure 2a). We compared prion seeding activity at 49 days post infection (dpi) with various doses of RML scrapie against the original protocol, which used 0.01% RML brain homogenate in a 1-hour soak (Figure 2b). PrP^Sc^ seeding activity showed a dose-dependent increase at 49 dpi (Supplementary Figure 3), with comparable seeding activity between inoculation methods at the same dose (0.01% RML brain homogenate). The post-culture inoculation approach was faster, less labor-intensive, and required substantially less inoculum (2 μL vs. 100 μL per slice) while preserving slice culture quality more effectively (Supplementary Figure 2c).

**Assessing nanobeads for prion inoculation**

Previous studies suggest that nanoparticles can enhance prion seeding efficiency^3^, prompting us to test their effect in POSCA. Cerebellar slice cultures were inoculated by applying 0.1% RML brain homogenate spiked with either silica (Si) or mesoporous (Me) nanospheres at dilutions ranging from 25 to 0.25 µg/mL, and accumulation of prion seeding activity was assessed at days 1, 28, and 56 days post inoculation (Supplementary Figure 3). We observed no differences between treatment groups, and so we concluded that spiking inoculum with nanoparticles did not affect prion infection under the conditions tested. Unless indicated otherwise, we inoculated slices via direct application of diluted brain homogenates, post-culture, without any additives in all further experiments.

**
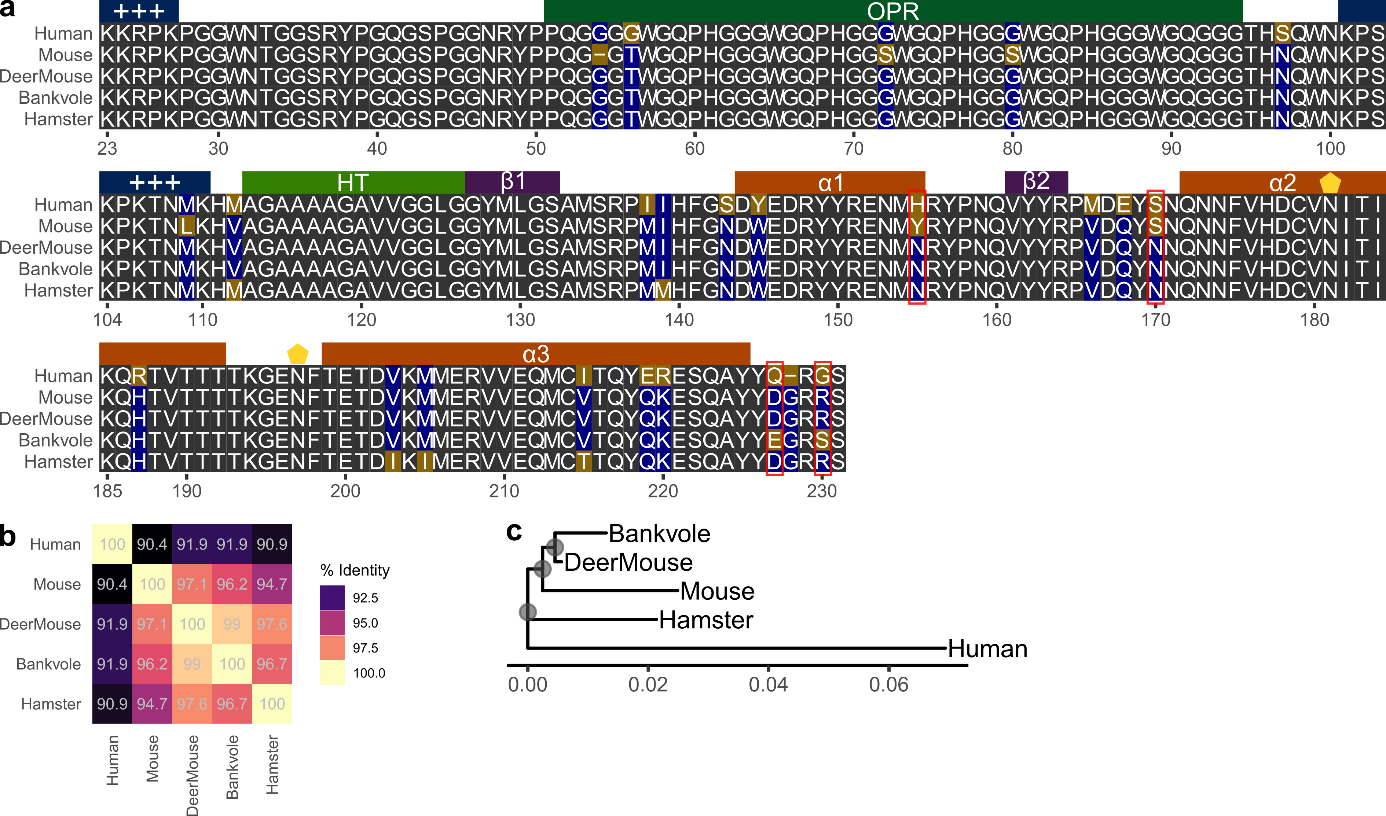
**

**Supplementary Figure 1. Amino acid sequence alignment of PrP across species. (a)** Multiple sequence alignment of PrP from humans, bank voles (*Myodes glareolus*), house mice (*Mus musculus*), and deer mice (*Peromyscus maniculatus*) generated using MUSCLE, with major structural features annotated: positively charged regions (+++), octapeptide repeats (OPR), hydrophobic tract (HT), β-sheets (β1 and β2), α-helices (α1, α2, α3), and N-glycosylation sites (N-glycans). Four polymorphisms associated with increased misfolding propensity in bank vole PrP (N155, N170, E227, and S230) are highlighted with red boxes. **(b)** Sequence similarity across species visualized through a sequence identity matrix. **(c)** Phylogenetic tree illustrating evolutionary relationships among PrP sequences from the four species.


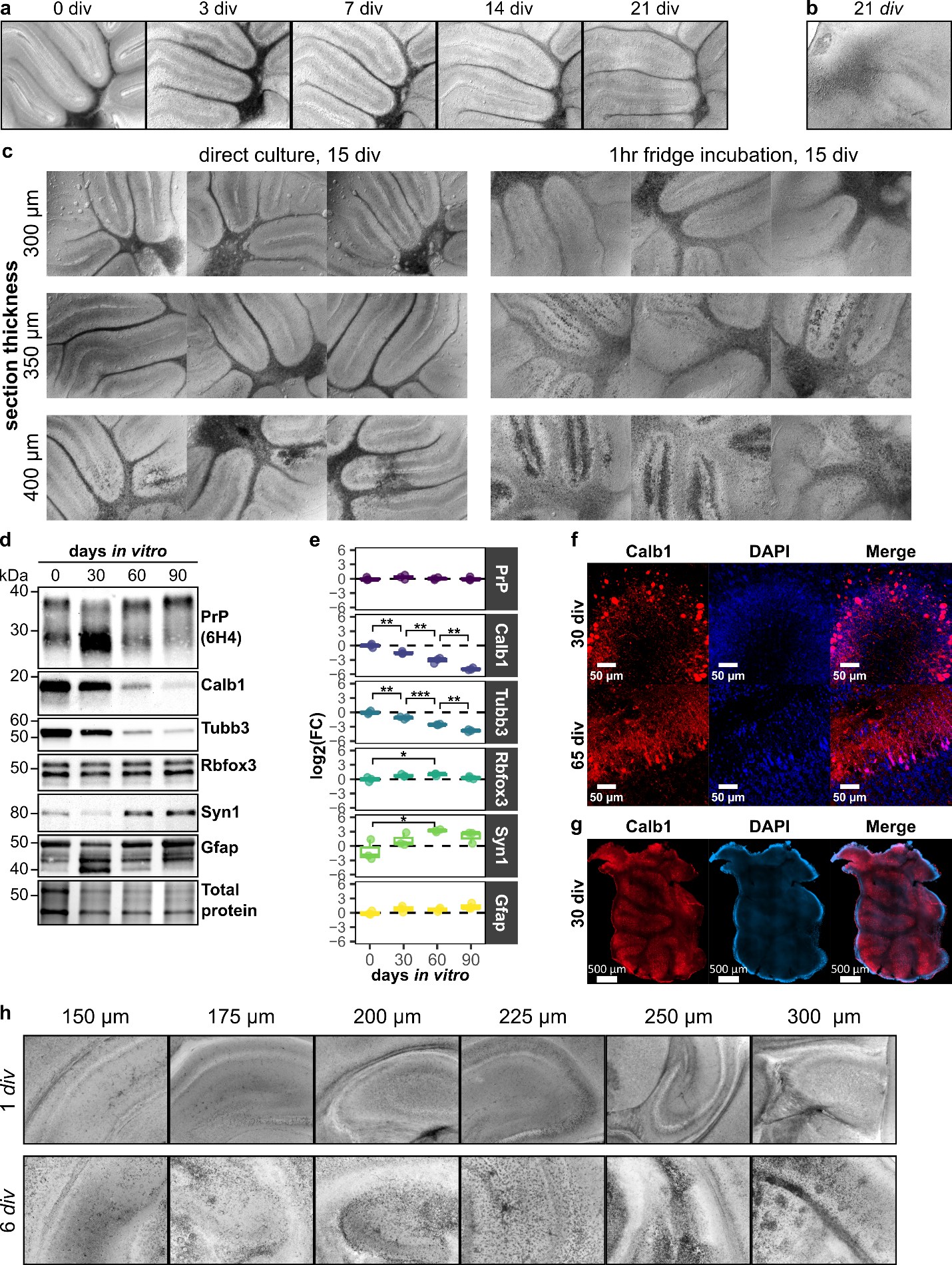


**Supplementary Figure 2. Assessment of CD1 brain slice culture viability. (a)** Phase contrast microscopy shows the well-preserved morphology of high-quality CD1 cerebellar slice cultures (300 µm thickness, directly cultured) maintained over 21 days *in vitro* (div). **(b)** For comparison, a phase contrast image of a poorly-preserved cerebellar section at 21 div is shown, highlighting differences in tissue quality. **(c)** Phase contrast microscopy demonstrates that reducing slice thickness improves cerebellar slice culture quality at 15 div. Images show slices directly cultured immediately after sectioning and following a 1-hour refrigeration period before culturing. **(d)** Western blot analysis confirmed the expression of key proteins, including PrP^C^, Calb1, Tubb3, Rbfox3, Syn1, and Gfap, in cerebellar slices cultured for 0, 30, 60, and 90 div (*n=3*). **(e)** Protein abundance was quantified at each timepoint, normalized to total protein signal, using ImageJ. *p*-values were calculated using one-way ANOVA (* p<0.05, ** p<0.01, *** p<0.001). **(f)** Immunofluorescence staining of Calb1 at 30 and 65 div confirmed the survival of Purkinje neurons in cerebellar slices via confocal microscopy. **(g)** Scanning fluorescence microscopy of an entire cerebellar section stained for Calb1 at 30 div also confirmed Purkinje neuron survival. **(h)** Phase contrast microscopy demonstrates that reducing slice thickness improves whole brain slice culture quality at 6 div.

**
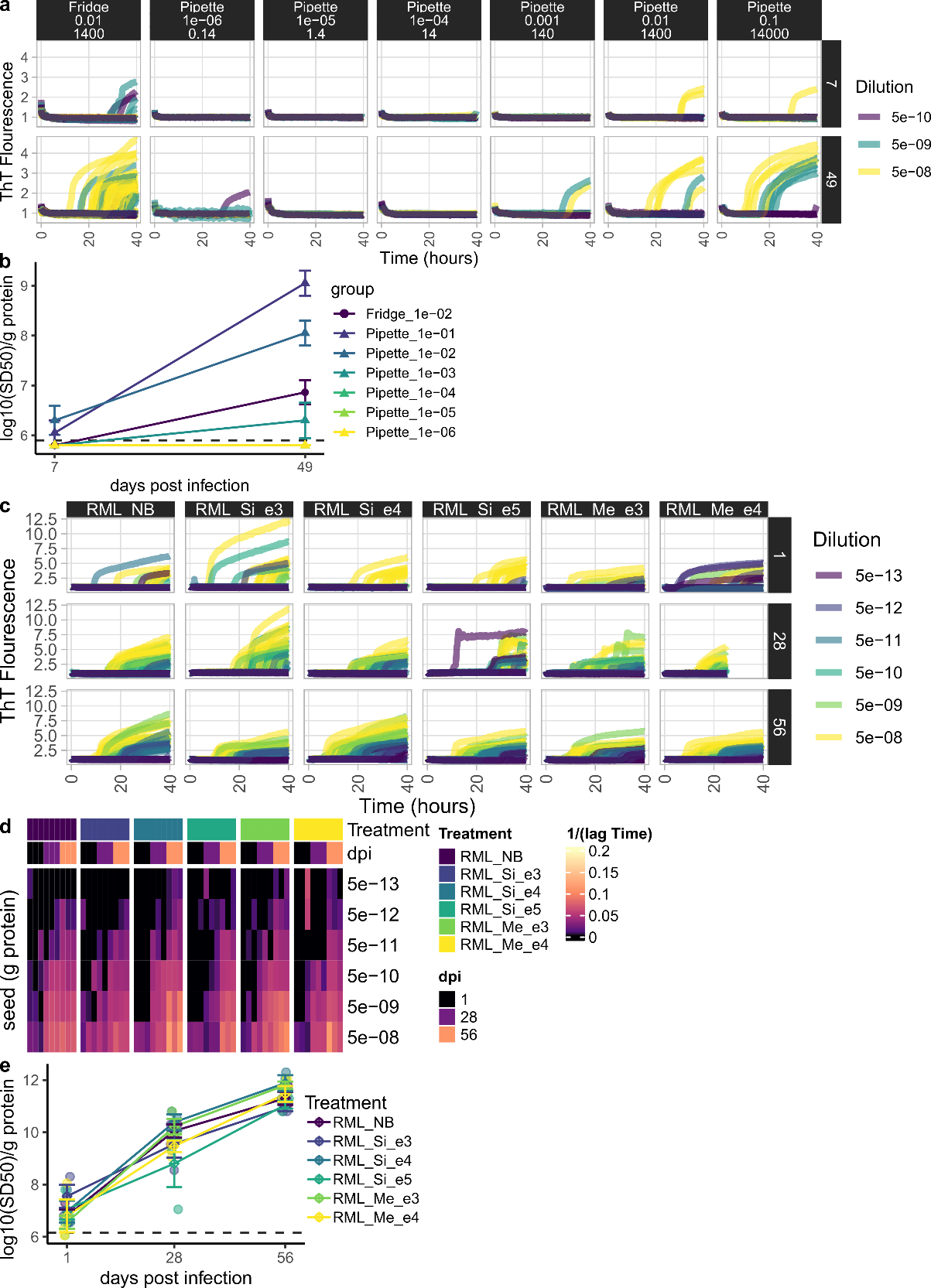
**

**Supplementary Figure 3. Optimizing inoculation of CD1 cerebellar slice cultures with RML scrapie (extended RT-QuIC data from Figures 2b and 2c). (a-b)** CD1 cerebellar slice cultures were inoculated with RML scrapie either by incubating slices with 0.01% RML brain homogenate for 1 hour prior to culturing or by applying RML brain homogenate directly onto slices after 1 day in culture at dilutions from 0.01% to 1x10^-6^%. **(a)** Thioflavin T (ThT) fluorescence curves and **(b)** SD50 measurements show RT-QuIC reactions seeded with lysates collected from slice cultures at 7 and 49 days post-inoculation. **(c-e)** To assess bead-assisted inoculation, cerebellar slices were seeded with 0.1% RML brain homogenate spiked with either silica or mesoporous nanobeads at dilutions of 1x10^-3^, 1x10^-4^, 1x10^-5^, or without beads. **(c)** ThT fluorescence curves, **(d)** inverse lag time (1/lag time) measurements, and **(e)** SD_50_ values are shown for RT-QuIC reactions seeded with slice culture lysates at 1, 28, and 56 days post-inoculation.

**
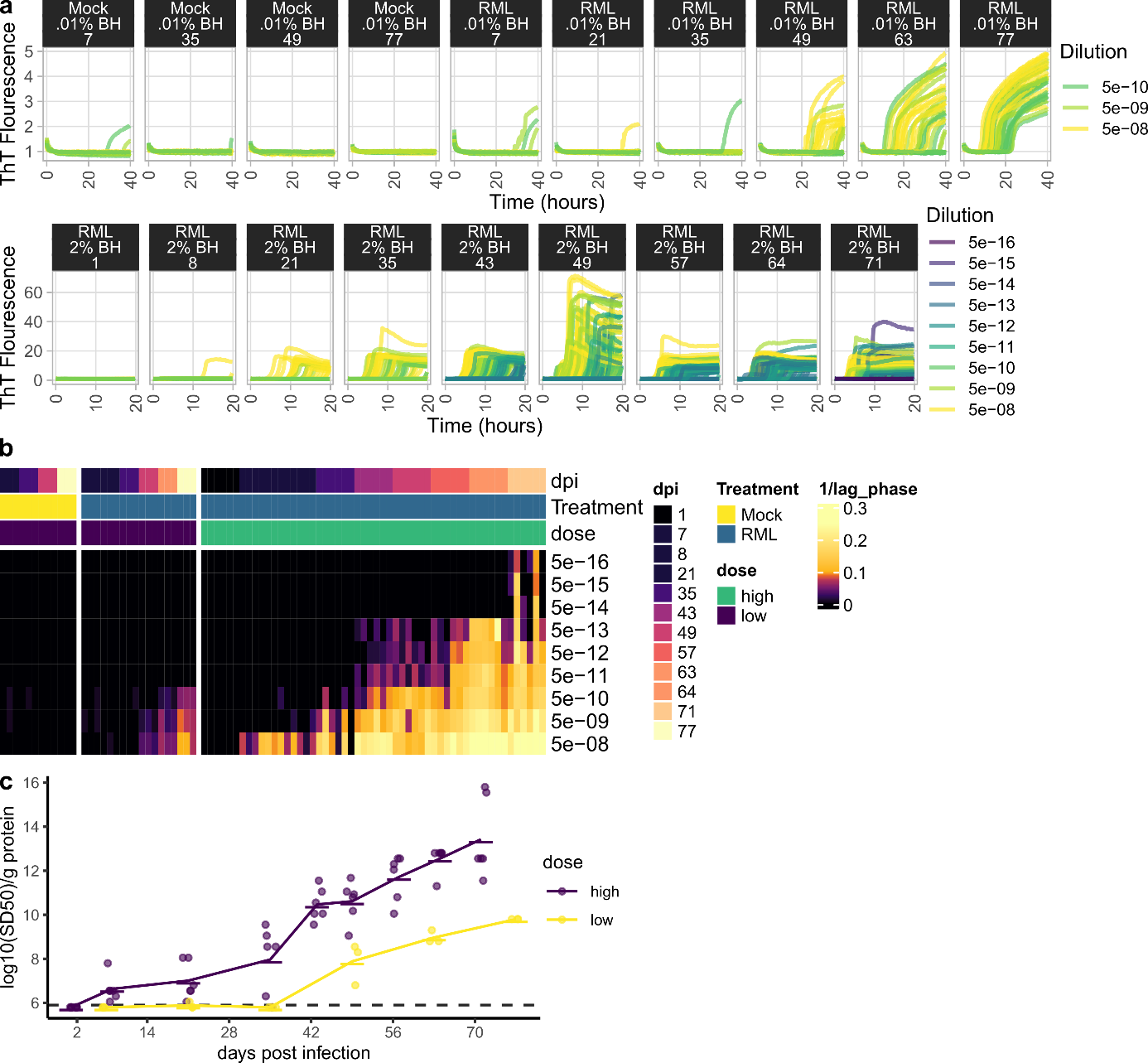
**

**Supplementary Figure 4. High- and low-dose RML scrapie challenge in CD1 cerebellar slice cultures (extended RT-QuIC data from Figure 2e).** CD1 cerebellar slice cultures were inoculated with either a high-dose (2% RML brain homogenate, applied directly after culturing) or a low-dose (0.01% RML brain homogenate, incubated pre-culture at 4 °C for 1 hour). A control group was treated with 0.01% non-infectious brain homogenate under the same pre-culture incubation conditions. Amyloid seeding activity was monitored at 14-day intervals, spanning 71–77 days post-inoculation. **(a)** Thioflavin T (ThT) fluorescence curves, **(b)** inverse lag time (1/lag time) measurements, and **(c)** SD_50_ values are presented for RT-QuIC reactions seeded with lysates from the cultures.

**
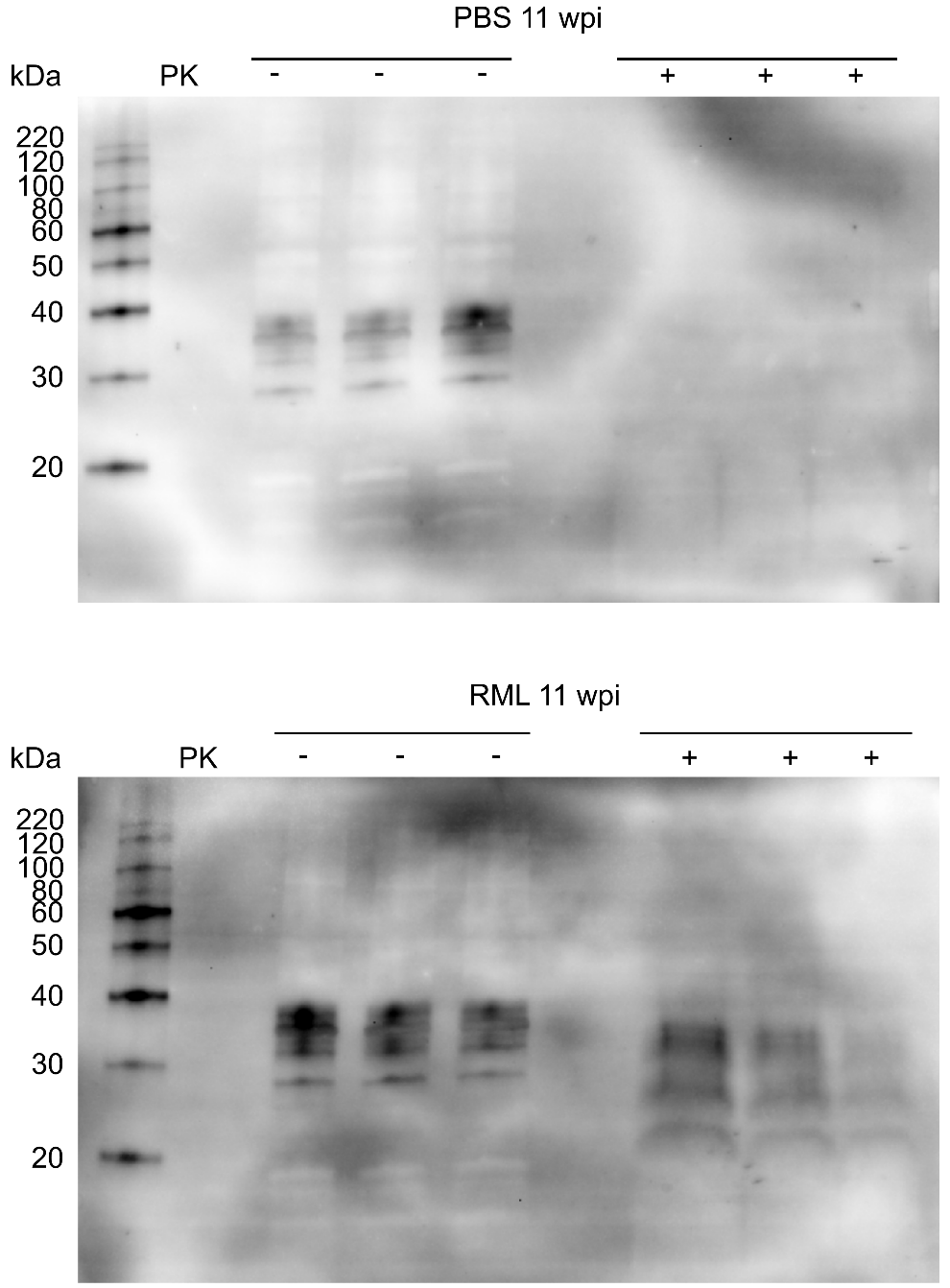
**

**Supplementary Figure 5. Assessment of PrP^RES^ in CD1 cerebellar slice cultures at 11-weeks following a standard-dose RML inoculation (uncropped western blot images from Figure 2f).** Slice culture lysate (100 µg) was digested with proteinase K (PK; 25 µg/mL) at 37 °C for 30 minutes, followed by western blotting for PrP using the 6H4 monoclonal antibody.

**
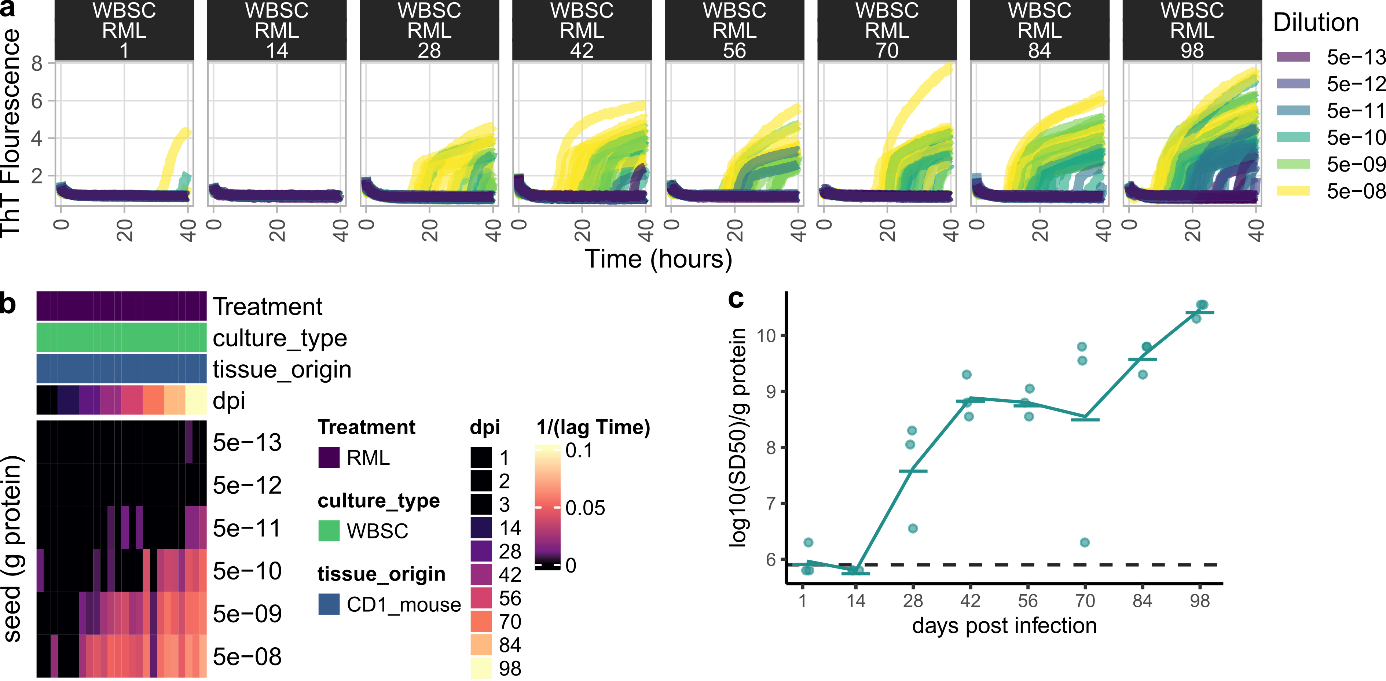
**

**Supplementary Figure 6. Monitoring amyloid seeding activity in CD1 whole brain slice cultures infected with RML scrapie (extended RT-QuIC data from Figure 2h).** CD1 whole brain slice cultures were inoculated with 0.01% RML brain homogenate, and amyloid seeding activity was tracked over 98 days post-inoculation at 14-day intervals. Shown are **(a)** Thioflavin T (ThT) fluorescence curves, **(b)** inverse lag time (1/lag time) measurements, and **(c)** SD50 values obtained from RT-QuIC reactions seeded with culture lysates.


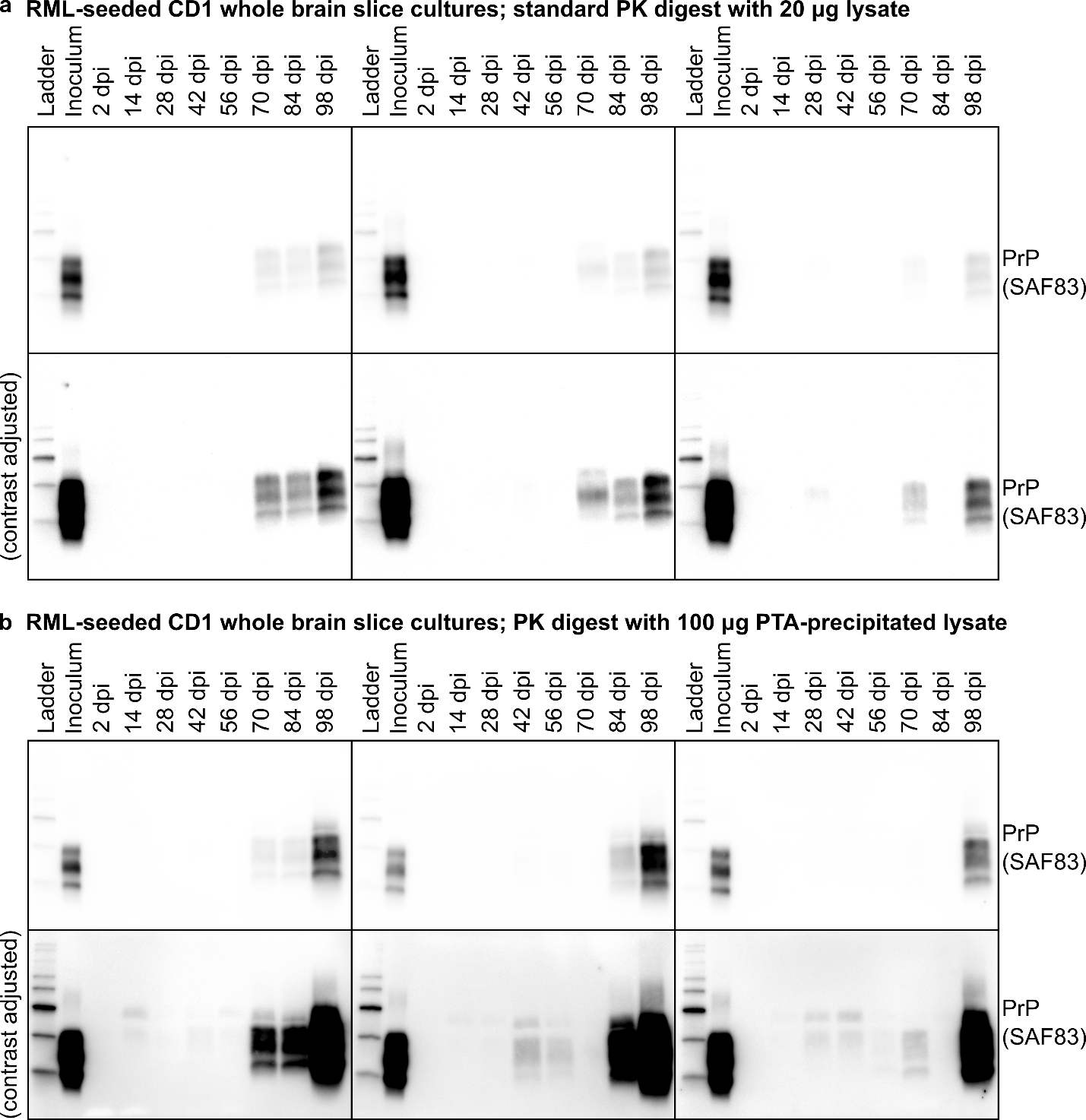


**Supplementary Figure 7. Assessment of PrP^RES^ in RML-seeded CD1 whole brain slice cultures (uncropped western blot images from Figure 2i).** CD1 whole brain slice (WBSC) cultures were inoculated with 0.01% RML brain homogenate, and proteinase K resistant PrP^Sc^ (PrP^RES^) was tracked over 98 days post-inoculation at 14-day intervals. **(a)** 20 µg of crude slice culture lysate, or **(b)** 100 µg of slice culture lysate precipitated with PTA, was digested with proteinase K (PK; 25 µg/mL) at 37 °C for 30 min and then western blotted for PrP using the SAF83 monoclonal antibody. RML inoculum was digested with proteinase K, and 1 µg was included for western blotting as a positive control.

**
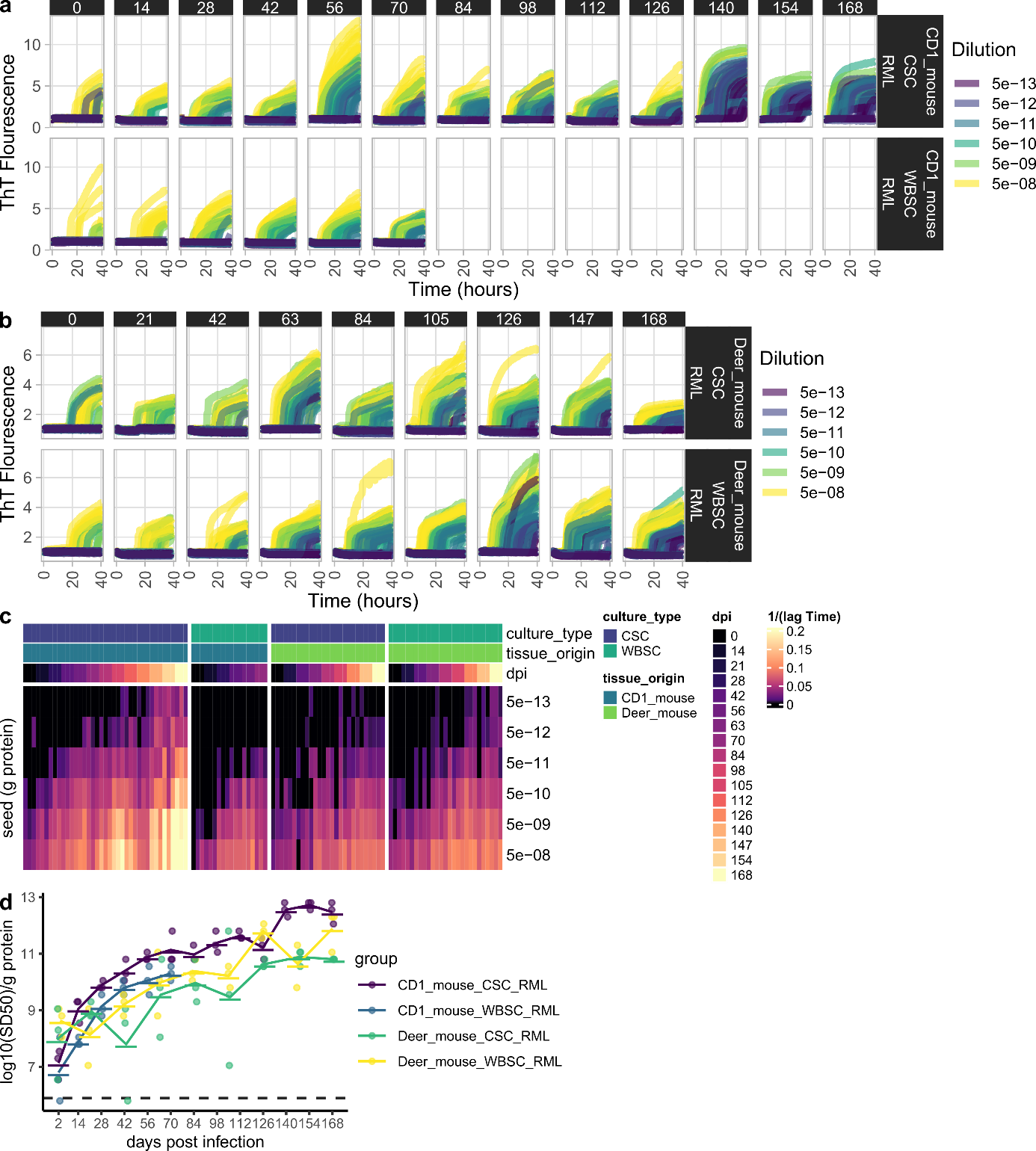
**

**Supplementary Figure 8. Amyloid seeding activity in CD1 and deer mouse slice cultures infected with RML scrapie (extended RT-QuIC data from Figure 3b).** Cerebellar (CSC) and whole brain (WBSC) slice cultures from CD1 and deer mice were inoculated with 0.1% RML brain homogenate. Amyloid seeding activity was assessed with RT-QuIC at 14-21 day intervals, spanning a 168-day infection time course. Shown are **(a)** Thioflavin T (ThT) fluorescence signal curves for CD1 and **(b)** deer mouse slice cultures, alongside **(c)** inverse lag time (1/lag time) measurements and (d) SD50 values.


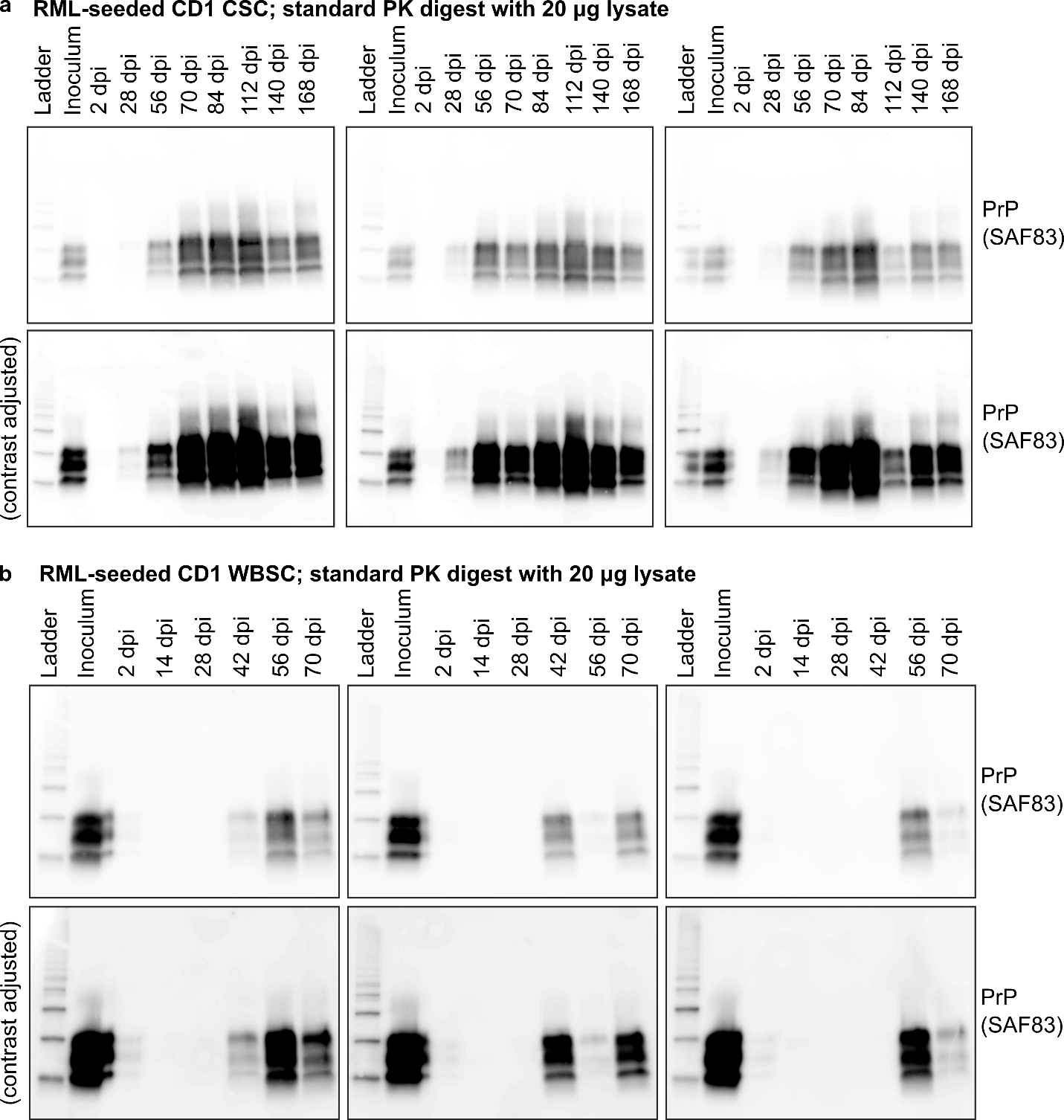


**Supplementary Figure 9. Assessment of PrP^RES^ in RML-seeded CD1 whole brain and cerebellar slice cultures (uncropped western blot images from Figure 3c).** CD1 **(a)** cerebellar (CSC) and **(b)** whole brain slice cultures (WBSC) were inoculated with 0.1% RML brain homogenate, and proteinase K resistant PrP^Sc^ (PrP^RES^) was tracked over 98 days post-inoculation at 14-day intervals. 20 µg of crude slice culture lysate was digested with proteinase K (PK; 25 µg/mL) at 37 °C for 30 min and then western blotted for PrP using the SAF83 monoclonal antibody. RML inoculum was digested with proteinase K, and 2 µg was included for western blotting as a positive control.


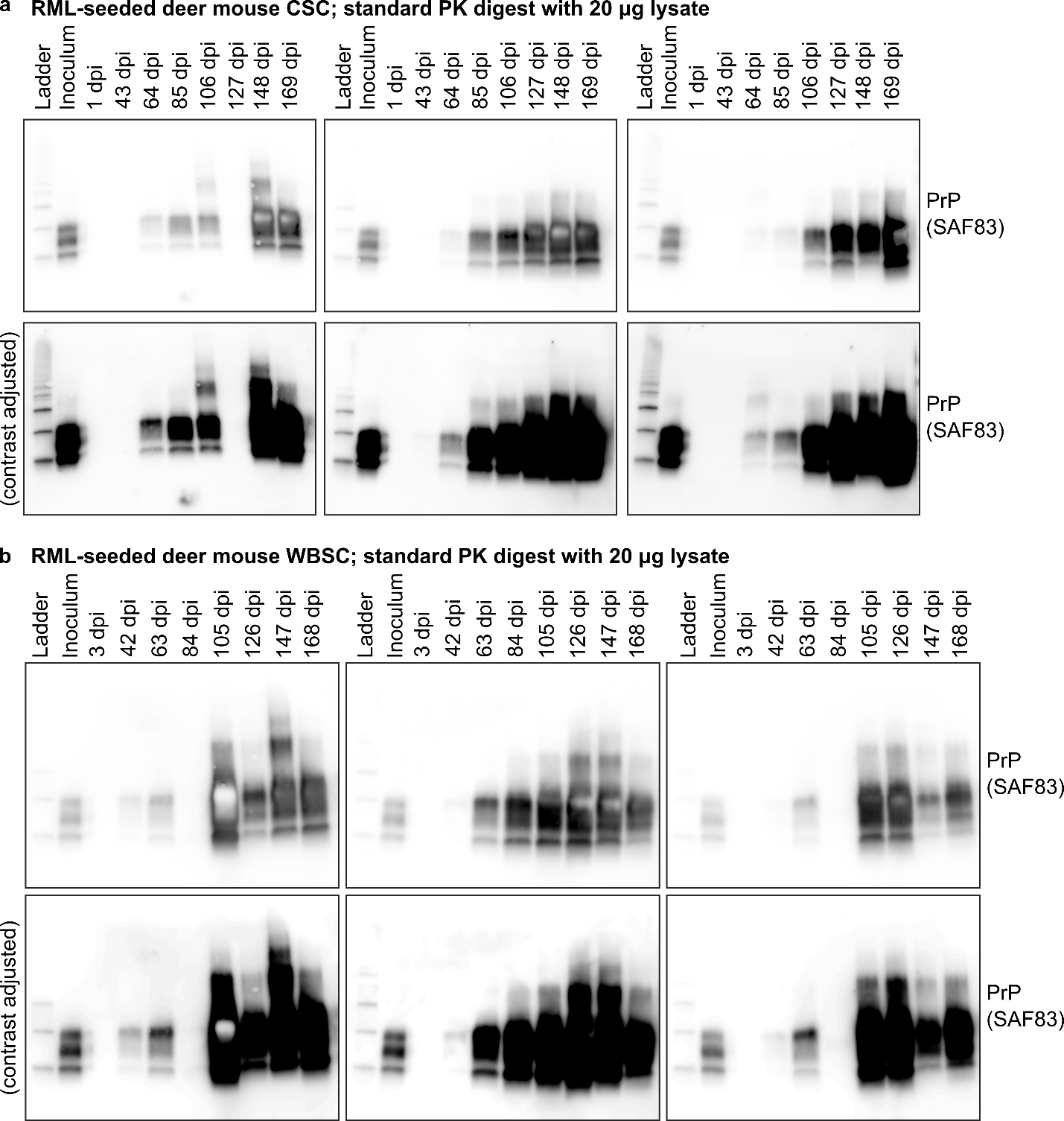


**Supplementary Figure 10. Assessment of PrP^RES^ in RML-seeded deer mouse whole brain and cerebellar slice cultures (uncropped western blot images from Figure 3c).** Deer mouse **(a)** cerebellar (CSC) and **(b)** whole brain slice cultures (WBSC) were inoculated with 0.1% RML brain homogenate, and proteinase K resistant PrP^Sc^ (PrP^RES^) was tracked over 98 days post-inoculation at 14-day intervals. 20 µg of crude slice culture lysate was digested with proteinase K (PK; 25 µg/mL) at 37 °C for 30 min and then western blotted for PrP using the SAF83 monoclonal antibody. RML inoculum was digested with proteinase K, and 2 µg was included for western blotting as a positive control.

**
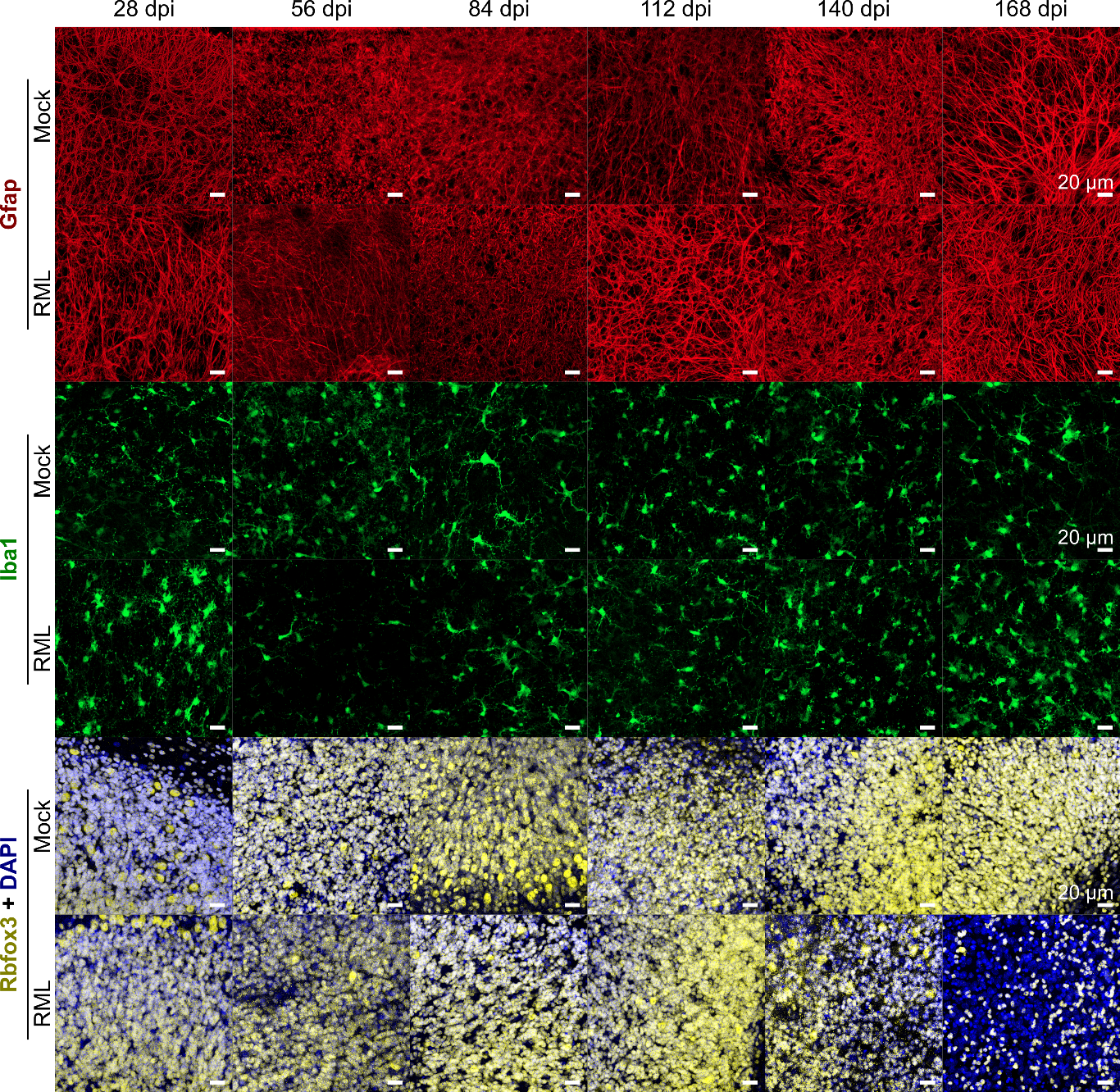
**

**Supplementary Figure 11. Prion neuropathology in CD1 cerebellar slice cultures.** CD1 cerebellar slice cultures were seeded with RML scrapie, or mock-infected, and analyzed using an immunofluorescence microscopy panel targeting Gfap (astrocytes), Iba1 (microglia) and Rbfox3 (neurons). Shown are representative maximum intensity projections of region-of-interest (ROI) images taken of prion and mock infected slice cultures at each timepoint spanning 168 days post infection (dpi).

**
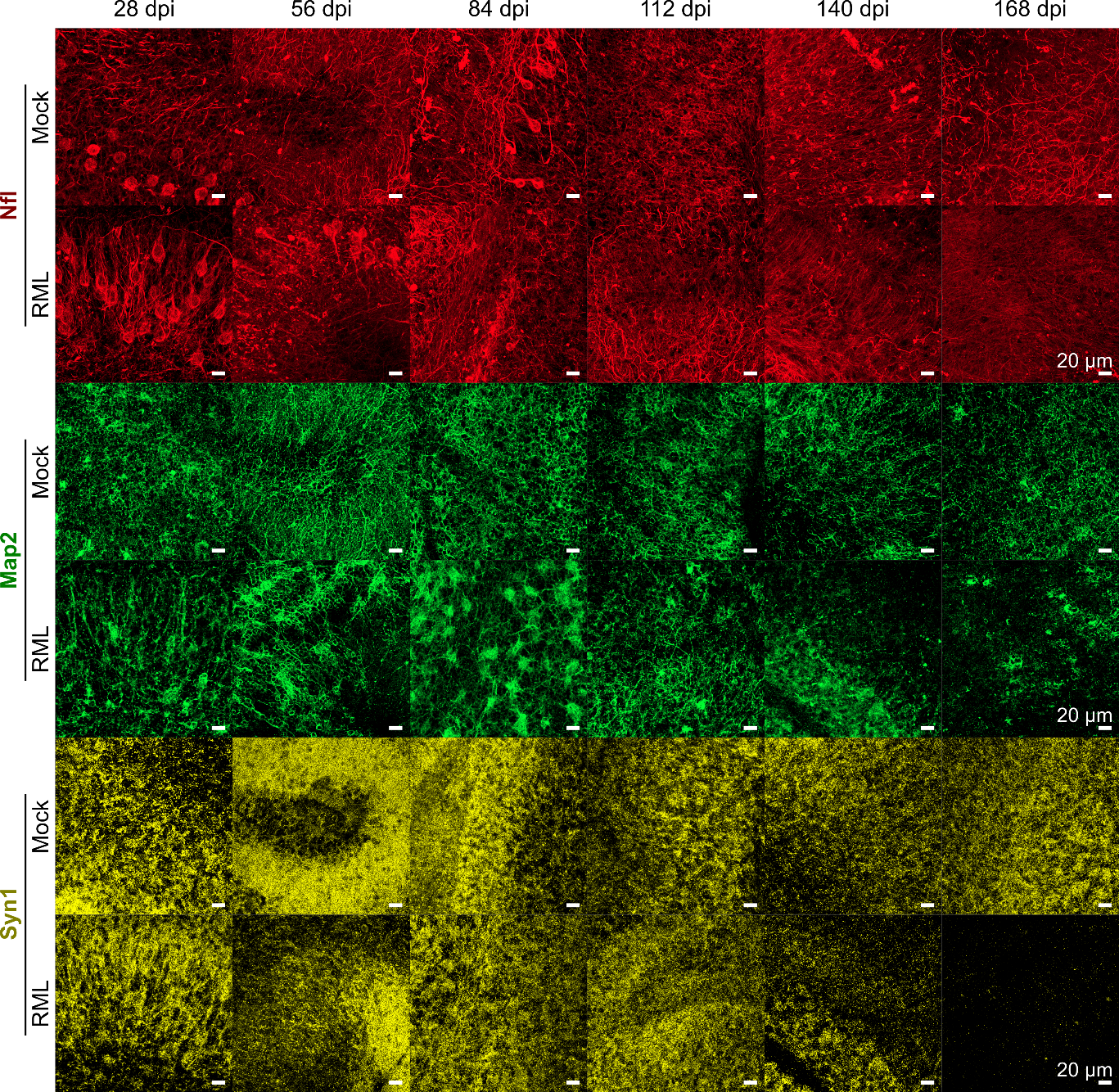
**

**Supplementary Figure 12. Prion synaptic toxicity in CD1 cerebellar slice cultures.** CD1 cerebellar slice cultures were seeded with RML scrapie, or mock-infected, and analyzed using an immunofluorescence microscopy panel targeting Nfl (neurofilaments), Map2 (dendrites), and Syn1 (synapses). Shown are representative maximum intensity projections of region-of-interest (ROI) images taken of prion and mock infected slice cultures at each timepoint spanning 168 days post infection (dpi).

**
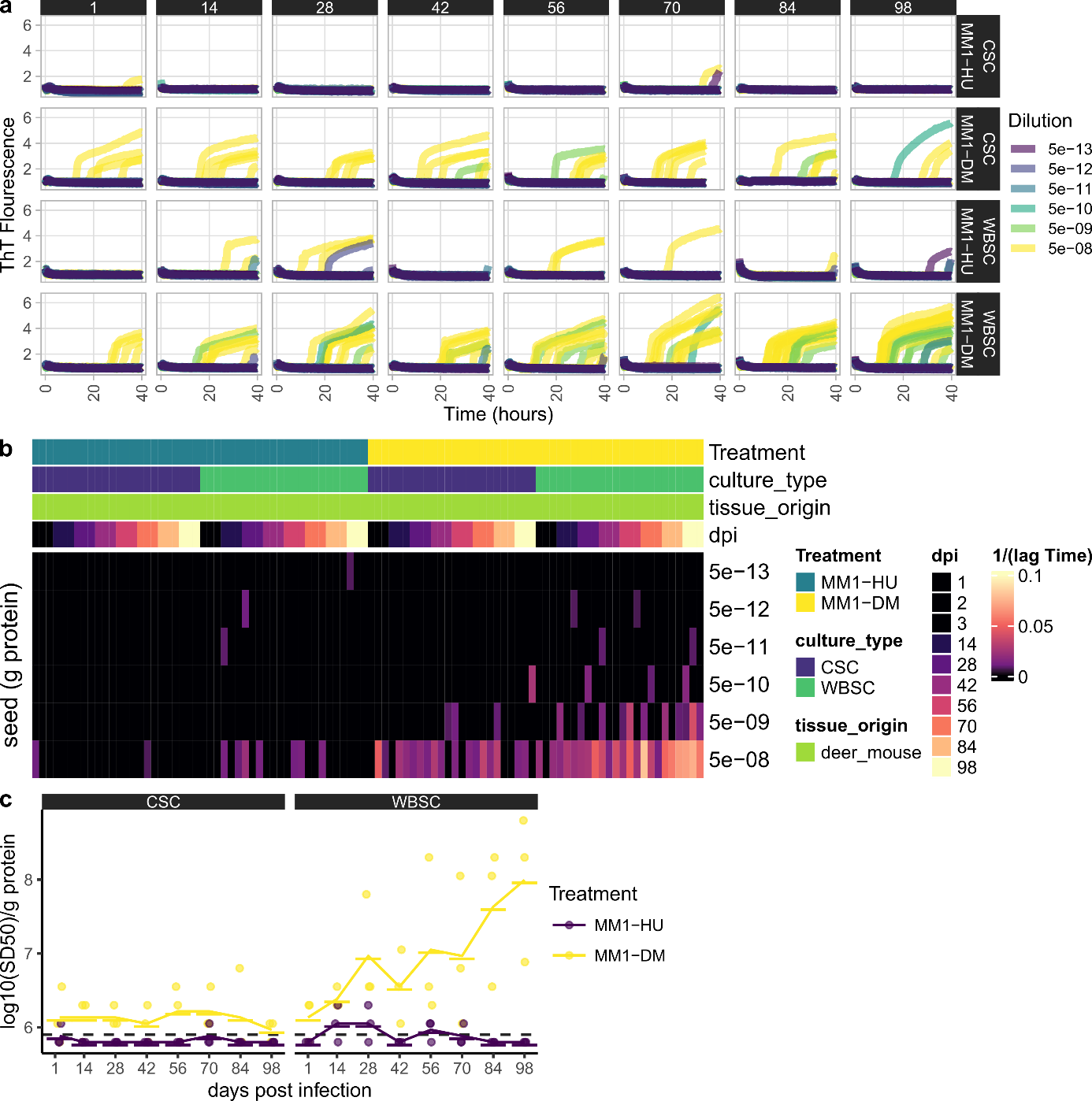
**

**Supplementary Figure 13. Amyloid seeding activity in deer mouse cerebellar and whole brain slice cultures infected with rodent-adapted and human sCJD MM1 prions (extended RT-QuIC data from Figure 5b).** Deer mouse cerebellar (CSC) and whole brain (WBSC) slice cultures were inoculated with human (MM1-HU) or mouse (MM1-DM, third passage) sCJD MM1 brain homogenates. Amyloid seeding activity in culture lysates was monitored at 14-day intervals, extending to 98 days post-infection. Presented are **(a)** Thioflavin T (ThT) fluorescence curves, **(b)** inverse lag time (1/lag time) measurements, and **(c)** SD_50_ values from RT-QuIC analyses.

**
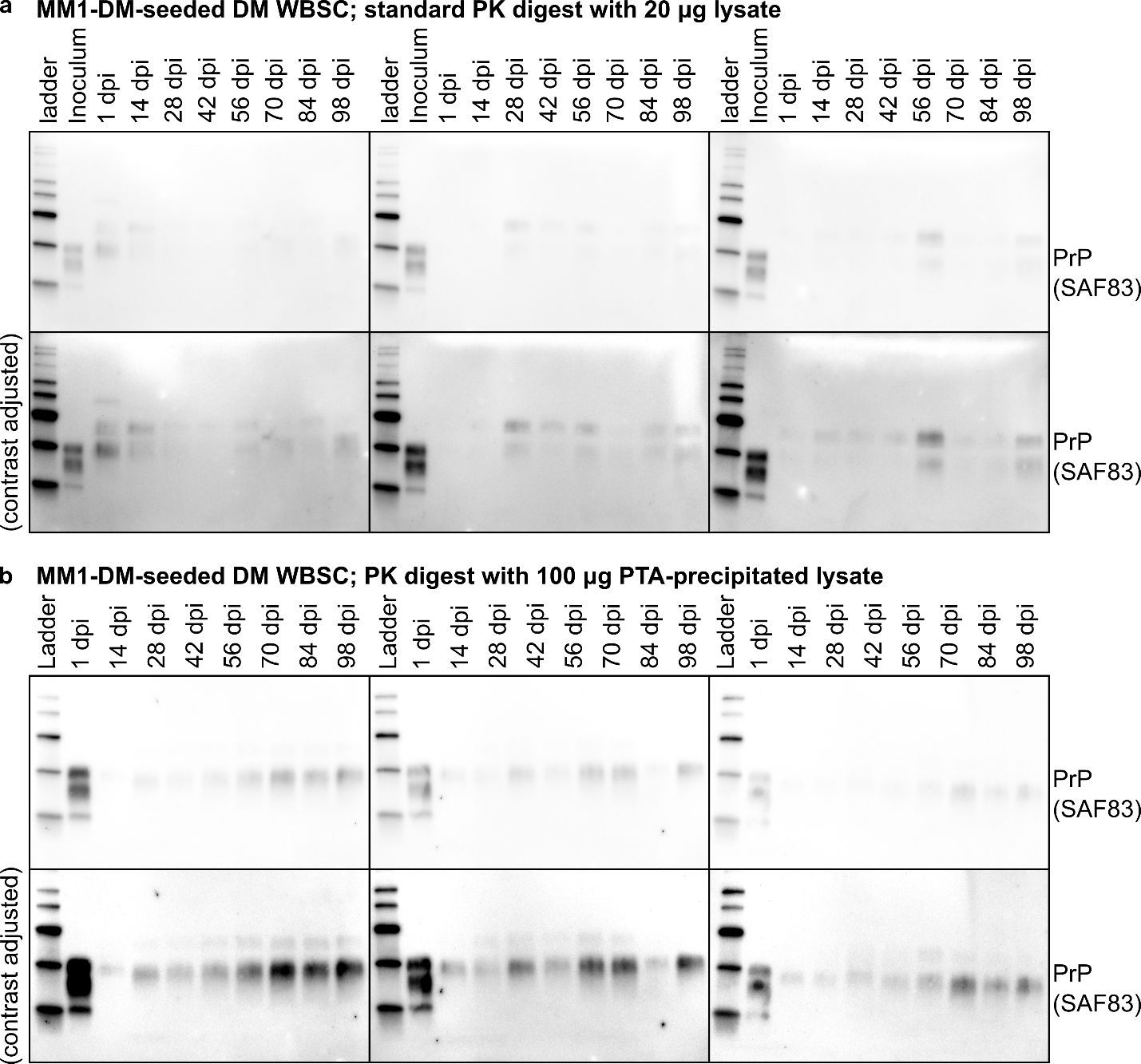
**

**Supplementary Figure 14. Assessment of PrP^RES^ in MM1-DM-seeded deer mouse whole brain slice cultures (uncropped western blot images from Figure 5c).** Deer mouse (DM) whole brain slice cultures (WBSC) were inoculated with 0.1% deer mouse adapted sCJD MM1 (MM1-DM) brain homogenate, and proteinase K resistant PrP^Sc^ (PrP^RES^) was tracked over 98 days post-inoculation at 14-day intervals. **(a)** 20 µg of crude slice culture lysate, or **(b)** 100 µg of slice culture lysate precipitated with PTA, was digested with proteinase K (PK; 25 µg/mL) at 37 °C for 30 min and then western blotted for PrP using the SAF83 monoclonal antibody. MM1-DM inoculum was digested with proteinase K, and 1 µg was included for western blotting as a positive control.


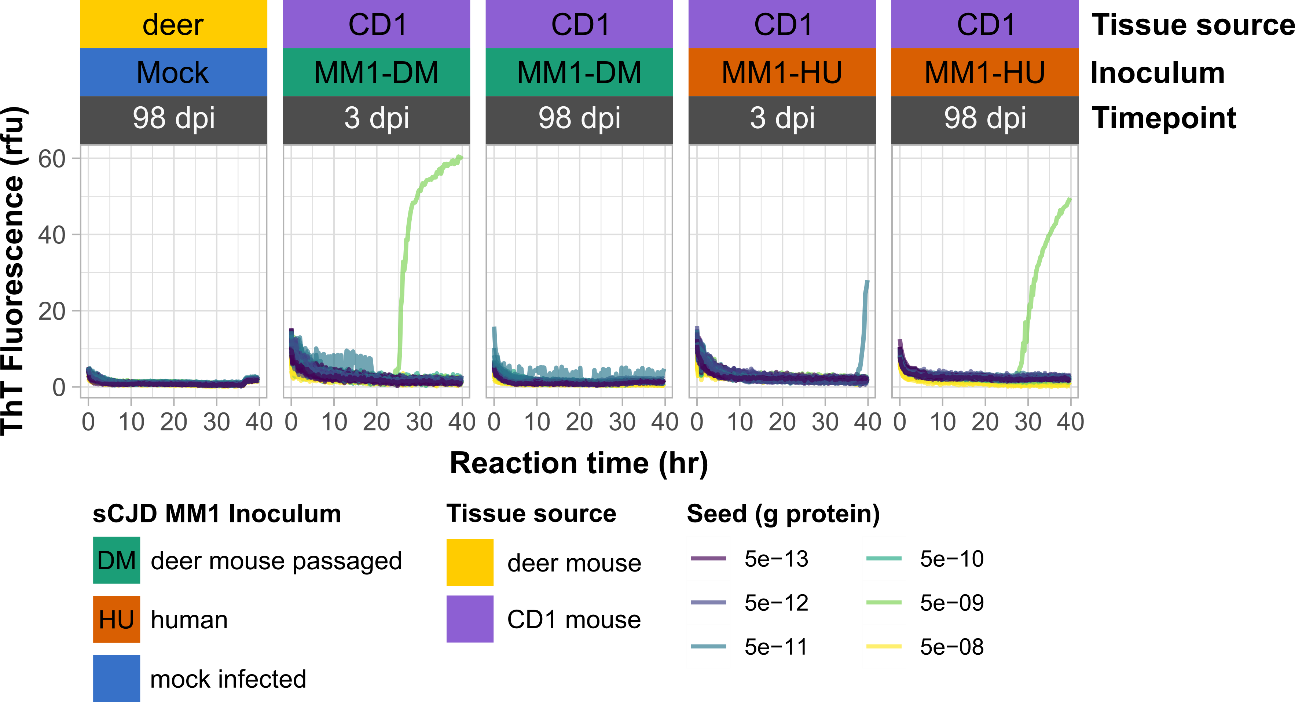


**Supplementary Figure 15. Lack of sCJD MM1 prion replication in CD1 cerebellar slice cultures.** CD1 cerebellar slice cultures were inoculated with 2 μL of 0.01% sCJD MM1 brain homogenate, using either the original human inoculum (MM1-HU) or a variant adapted through three passages in deer mice (MM1-DM). A non-infectious brain homogenate (Mock) served as a control. PrP^Sc^ seeding activity was measured at 0 and 14 weeks post-inoculation (*n=3*) via RT-QuIC, with Thioflavin T (ThT) fluorescence plotted over RT-QuIC reaction time.

**
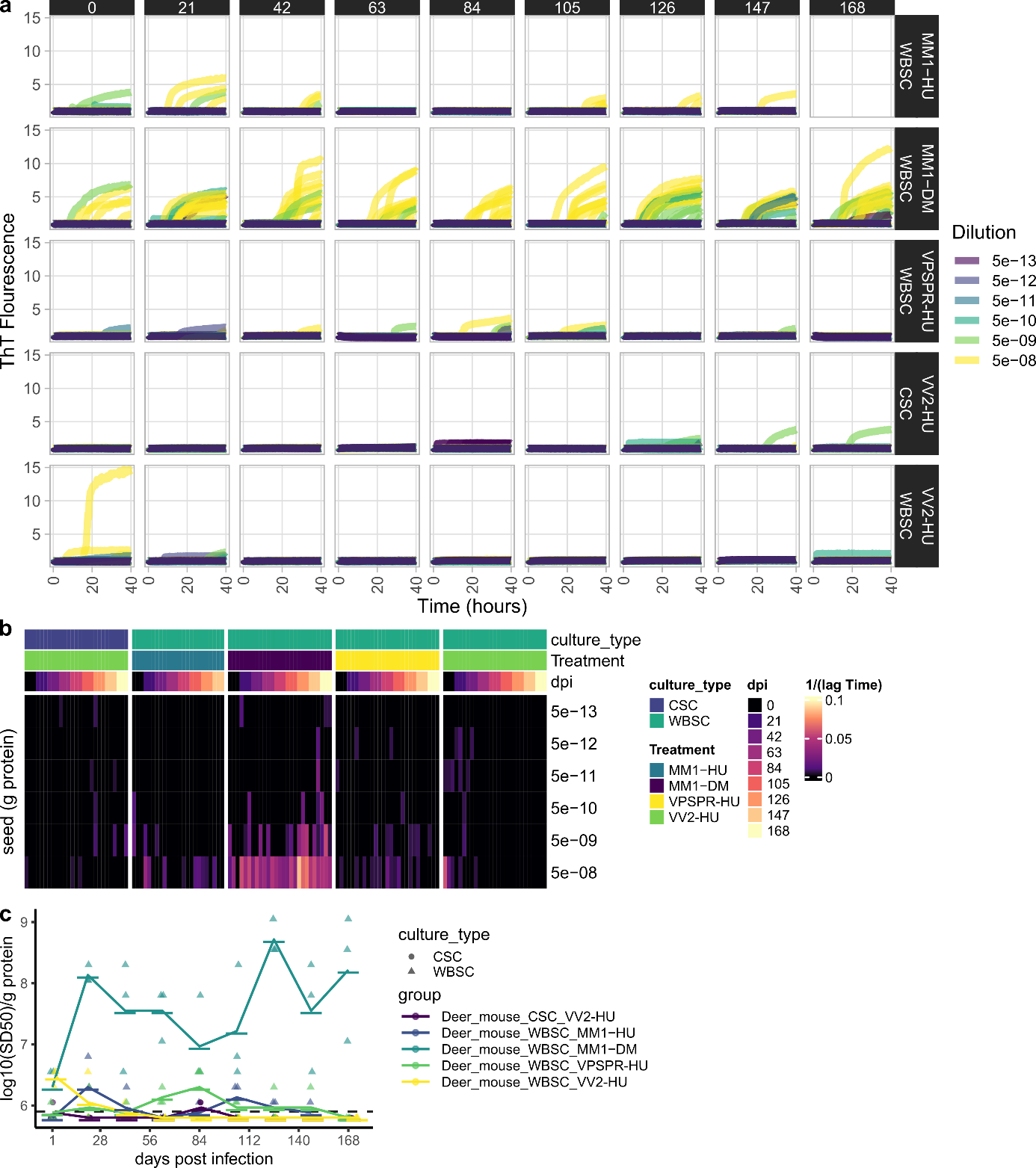
**

**Supplementary Figure 16. Amyloid seeding activity in deer mouse slice cultures inoculated with various CJD brain homogenates (extended RT-QuIC data from Figure 5e).** Deer mouse cerebellar slice cultures (CSC) were inoculated with brain homogenate from human sCJD VV2 (VV2-HU). Deer mouse whole brain slice cultures (WBSC) were inoculated with either human sCJD MM1 (MM1-HU), deer mouse-adapted sCJD MM1 (MM1-DM, following three passages), human sCJD VV2, or VPSPR (VPSPR-HU) brain homogenates. Amyloid seeding activity in culture lysates was assessed at 21-day intervals, extending to 168 days post-infection. Shown are **(a)** Thioflavin T (ThT) fluorescence curves, **(b)** inverse lag time (1/lag time) measurements, and **(c)** SD_50_ values from RT-QuIC analyses.


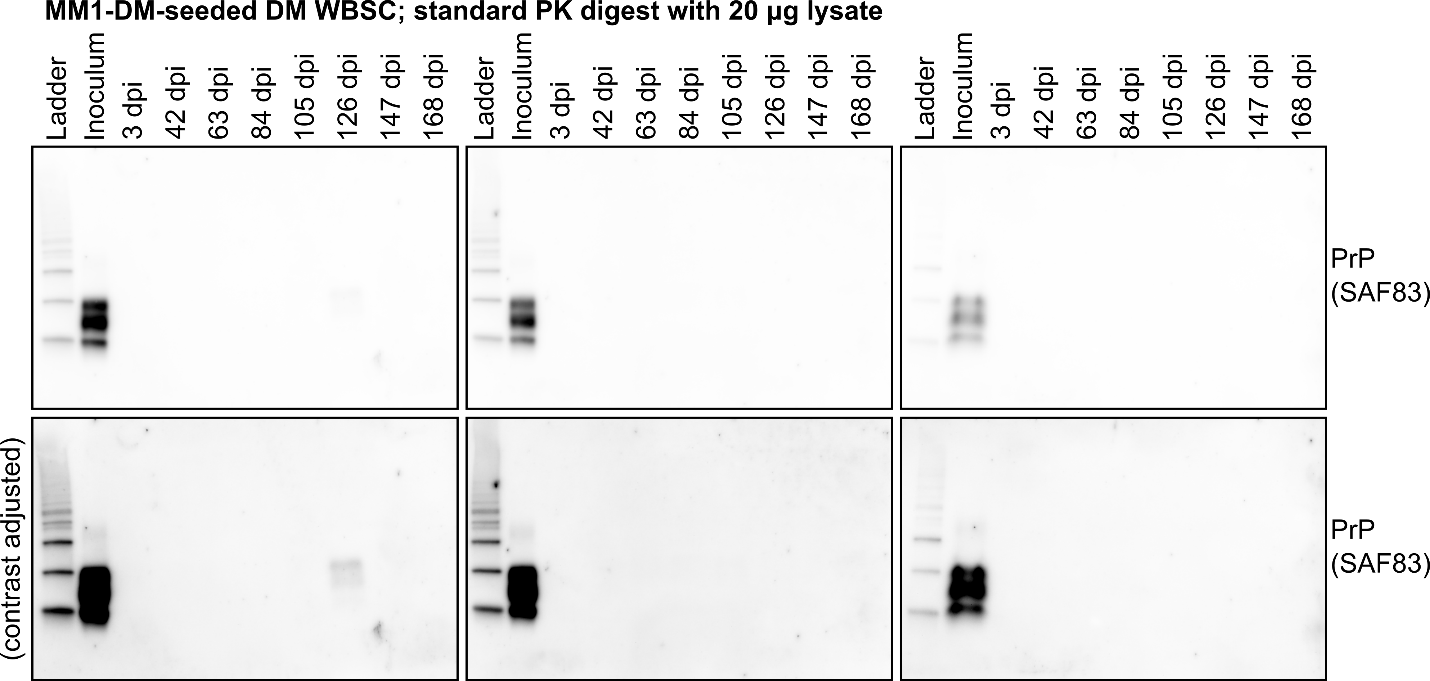


**Supplementary Figure 17. Assessment of PrP^RES^ in MM1-DM-seeded deer mouse whole brain slice cultures (uncropped western blot images from Figure 5f).** Deer mouse (DM) whole brain slice cultures (WBSC) were inoculated with 1% deer mouse adapted sCJD MM1 (MM1-DM) brain homogenate, and proteinase K resistant PrP^Sc^ (PrP^RES^) was tracked over 168 days post-inoculation at 21-day intervals. 20 µg of crude slice culture lysate was digested with proteinase K (PK; 25 µg/mL) at 37 °C for 30 min and then western blotted for PrP using the SAF83 monoclonal antibody. MM1-DM inoculum was digested with proteinase K, and 1 µg was included for western blotting as a positive control.


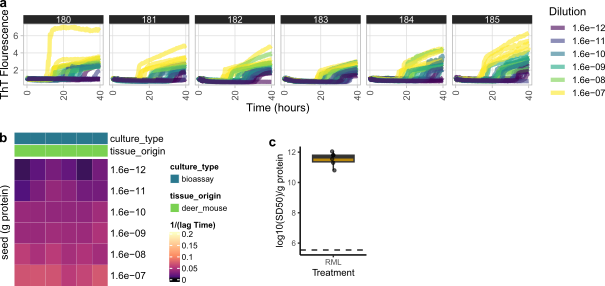


**Supplementary Figure 18. Prion seeding activity in brain homogenates from deer mice at the clinical endpoint of RML scrapie infection (extended RT-QuIC data from Figure 6d).** Deer mice were intracranially inoculated with RML brain homogenate and sacrificed at the clinical endpoint. RT-QuIC was used to measure amyloid seeding activity in deer mouse brain homogenates. Shown are **(a)** Thioflavin T (ThT) fluorescence curves, **(b)** inverse lag time (1/lag time) measurements, and **(c)** SD_50_ values from RT-QuIC analyses.

**
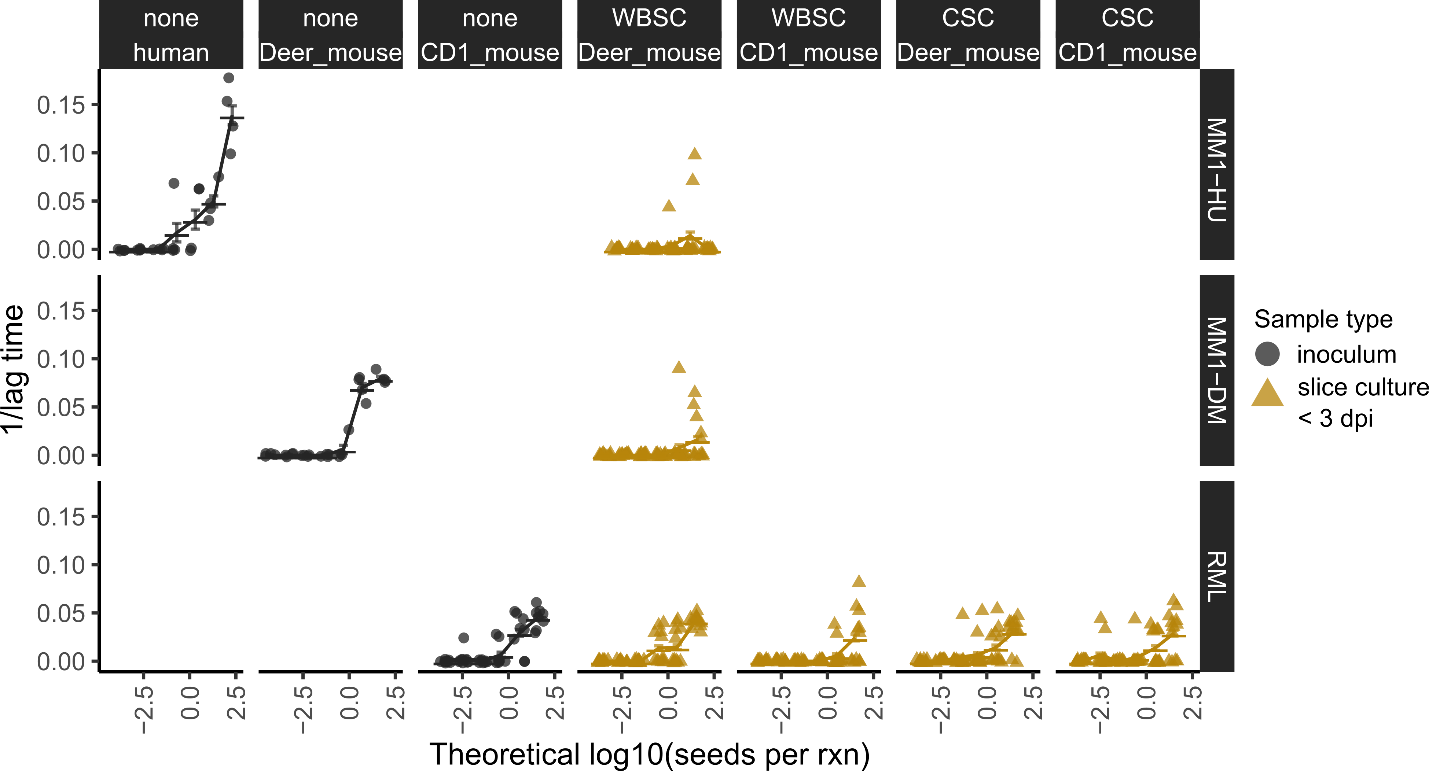
**

**Supplementary Figure 19. Residual inoculum prion seeding activity stratified by culture type and species (extended RT-QuIC data from Figure 7e).** Residual inoculum seeding activity was quantified slice cultures samples collected within the first 3 dpi following challenge with MM1-HU, MM1-DM, and RML using RT-QuIC. Seeding activity was assessed by plotting inverse lag time (1/lag time) measurements in RT-QuIC against the theoretical seed count per reaction. To compare the observed with the expected residual inoculum signal, the same analysis was applied to the diluted brain homogenates used for inoculation. Residual inoculum data is stratified by culture type (cerebellar [CSC] and whole brain slice cultures [WBSC]) and species (CD1 and deer mice).


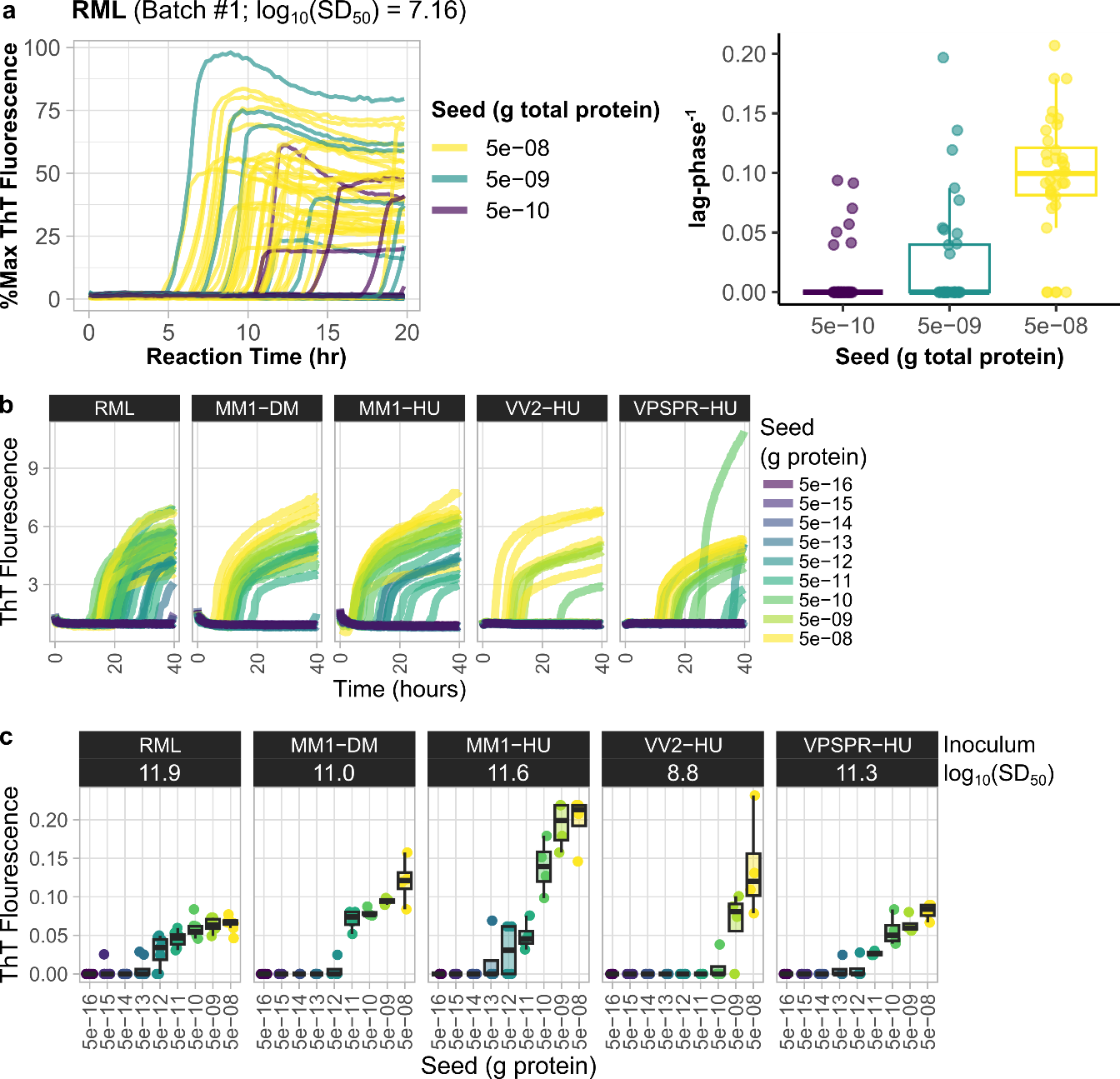


**Supplementary Figure 20. Prion seeding activity in inocula used in this study.** RT-QuIC was used to measure amyloid seeding activity in the different inocula used for **(a)** the initial optimization of RML infection in cerebellar slice cultures, and **(b-c)** sCJD challenges of deer mouse cerebellar and whole brain slice cultures.

**References**

1. Falsig, J. & Aguzzi, A. The prion organotypic slice culture assay--POSCA. *Nat Protoc* **3**, 555–562 (2008).

2. Uggerud, I. M., Kråkenes, T., Hirai, H., Vedeler, C. A. & Schubert, M. Development and Optimization of a Multilayer Rat Purkinje Neuron Culture. *Cerebellum (London, England)* (2023) doi:10.1007/S12311-022-01510-4.

3. Christenson, P. R., Li, M., Rowden, G., Larsen, P. A. & Oh, S.-H. Nanoparticle-Enhanced RT-QuIC (Nano-QuIC) Diagnostic Assay for Misfolded Proteins. *Nano Lett* **23**, 4074–4081 (2023).
